# Short bowel syndrome results in increased gene expression associated with proliferation, inflammation, bile acid synthesis and immune system activation: RNA sequencing a zebrafish SBS model

**DOI:** 10.1186/s12864-016-3433-4

**Published:** 2017-01-25

**Authors:** Kathy A. Schall, Matthew E. Thornton, Mubina Isani, Kathleen A. Holoyda, Xiaogang Hou, Ching-Ling Lien, Brendan H. Grubbs, Tracy C. Grikscheit

**Affiliations:** 1 Division of Pediatric Surgery and Developmental Biology and Regenerative Medicine, Saban Research Institute, Children’s Hospital Los Angeles and USC Keck School of Medicine, Los Angeles, CA 90027 USA; 2 Department of Obstetrics and Gynecology, Division of Maternal Fetal Medicine, Saban Research Institute, Children’s Hospital Los Angeles and USC Keck School of Medicine, Los Angeles, CA 90027 USA; 3 Division of Cardiothoracic Surgery, Saban Research Institute, Children’s Hospital Los Angeles and USC Keck School of Medicine, Los Angeles, CA 90027 USA; 4 Department of Surgery, Children’s Hospital Los Angeles, 4650 Sunset Blvd, Mailstop 100, Los Angeles, CA 90027 USA

**Keywords:** Short bowel syndrome, RNA sequencing, Intestinal resection, Inflammation, Cell proliferation, Innate and adaptive immunity

## Abstract

**Background:**

Much of the morbidity associated with short bowel syndrome (SBS) is attributed to effects of decreased enteral nutrition and administration of total parenteral nutrition (TPN). We hypothesized that acute SBS alone has significant effects on gene expression beyond epithelial proliferation, and tested this in a zebrafish SBS model.

**Methods:**

In a model of SBS in zebrafish (laparotomy, proximal stoma, distal ligation, *n* = 29) or sham (laparotomy alone, *n* = 28) surgery, RNA-Seq was performed after 2 weeks. The proximal intestine was harvested and RNA isolated. The three samples from each group with the highest amount of RNA were spiked with external RNA controls consortium (ERCC) controls, sequenced and aligned to reference genome with gene ontology (GO) enrichment analysis performed. Gene expression of *ctnnb1*, *ccnb1*, *ccnd1*, *cyp7a1a*, *dkk3*, *ifng1*-*2*, *igf2a*, *il1b*, *lef1*, *nos2b*, *saa1*, *stat3*, *tnfa* and *wnt5a* were confirmed to be elevated in SBS by RT-qPCR.

**Results:**

RNA-seq analysis identified 1346 significantly upregulated genes and 678 significantly downregulated genes in SBS zebrafish intestine compared to sham with Ingenuity analysis. The upregulated genes were involved in cell proliferation, acute phase response signaling, innate and adaptive immunity, bile acid regulation, production of nitric oxide and reactive oxygen species, cellular barrier and coagulation. The downregulated genes were involved in folate synthesis, gluconeogenesis, glycogenolysis, fatty-acid oxidation and activation and drug and steroid metabolism. RT-qPCR confirmed gene expression differences from RNA-Sequencing.

**Conclusion:**

Changes of gene expression after 2 weeks of SBS indicate complex and extensive alterations of multiple pathways, some previously implicated as effects of TPN. The systemic sequelae of SBS alone are significant and indicate multiple targets for investigating future therapies.

**Electronic supplementary material:**

The online version of this article (doi:10.1186/s12864-016-3433-4) contains supplementary material, which is available to authorized users.

## Background

Short bowel syndrome (SBS) and intestinal failure occur after surgical resection of large amounts of small intestine, which is a necessary response to multiple congenital anomalies, newborn surgical emergencies or trauma. The incidence of SBS is almost double the cumulative incidence of all invasive childhood cancers and has a 30% 5-year mortality [1, 2]. The usual treatment for SBS is the administration of intravenous (IV) nutrition because there is inadequate available intestinal surface area to absorb sufficient nutrition. A reduction of just 10% of US patients requiring home IV nutrition for SBS would result in estimated savings of $780,000,000 [3]. The patients who wean off of IV nutrition for SBS are able to do so because the remainder of their intestine undergoes adaptation. In intestinal adaptation, the epithelial surface area markedly increases with taller villi and deeper crypts, which results in a gain of available cell surfaces to absorb nutrition. For this reason, SBS has been particularly understood as a problem of diminished nutritional absorption, and the epithelial response has been much more studied than the in vivo intestine as a whole. But systemic effects beyond epithelial responses to nutrition such as inflammation, infection, cholestasis, hepatic fibrosis, electrolyte abnormalities and catheter related infections are observed in patients with SBS [4–6]. These systemic effects have been attributed to some of the treatment therapies as opposed to the actual disease process, such as the association of liver fibrosis to the administration of IV nutrition.

The systemic effects resulting from SBS have not been extensively studied. However, isolated SBS was found to create a systemic pro-inflammatory state that is magnified by sepsis independent of TPN [4]. Given that effects of SBS on gene expression in cell types outside of the epithelium have not been intensively studied, we evaluated matched samples of zebrafish intestine with and without SBS that was established 2 weeks prior to harvest, a model we previously validated [7], with RNA sequencing. This SBS model demonstrated increased villus epithelial perimeter, a measure of the exuberant epithelial expansion that accompanies intestinal adaptation in human SBS, as well as increased proliferation with more BrdU+ cells identified at 2 weeks after SBS surgery. The significant increase in BrdU+ cells was identified at 2 weeks in the SBS group, but is no longer statistically significant by the 4-week timepoint. The 2-week timepoint was chosen for these experiments to capture data at a known timepoint of cellular proliferation during adaptation in SBS. This analysis identified 1346 upregulated genes and 678 downregulated genes in SBS zebrafish intestine compared to sham-operated fish, with key genes confirmed by PCR. The upregulated genes were involved in cell proliferation, acute phase response signaling, innate and adaptive immunity, bile acid regulation, production of nitric oxide and reactive oxygen species, cellular barrier and coagulation. The downregulated genes were involved in folate synthesis, gluconeogenesis, glycogenolysis, fatty-acid oxidation and activation and drug and steroid metabolism.

## Methods

All protocols were approved by Children’s Hospital Los Angeles animal care facility and IACUC.

### Generation of SBS and sham zebrafish

We previously reported a zebrafish SBS model in which the intestine is resected at a reproducible site analogous to a human jejunostomy [7]. To generate SBS for RNA-seq analysis, we followed this established protocol, and adult male wild-type Ekk zebrafish were grouped into either SBS surgery (*n* = 29) or sham (*n* = 28) groups. Zebrafish were housed and handled in accordance with our approved animal protocol, and maintained in tank water changed every other day and pH balanced. Health checks were performed at these times and more frequently just after surgery, in accordance with our protocol. 3 from each group were harvested for RNA sequencing at 2 weeks and the remaining fish were harvested for evaluation by histology or RT-qPCR. The number of replicates was determined by a pilot experiment and statistical power analysis. False discovery rate adjusted *p*-values (Benjamini-Hochberg) were generated by edgeR software after a general logistic model fit and in comparison to a negative binomial distribution of the same size.

Briefly, the zebrafish were anesthetized with 0.02% tricaine and placed on an operating sponge under the stereomicroscope (Olympus SZX9). A ventral laparotomy was made anterior to the anal fins, the liver swept cephalad and the proximal loop of intestine brought out. The distal intestine at the junction of segment 3 and 4 (S3/S4) was suture ligated with 10–0 monofilament polypropylene and the proximal intestine was tacked to the abdominal wall at the junction of segment 1 and 2 (S1/S2). The mid-portion (S2/S3) was removed and the abdominal contents were placed back into the abdominal cavity, leaving behind a proximal functional ostomy. The sham operation consisted of a ventral laparotomy with no bowel manipulation.

### Weight measurement

The zebrafish were weighed weekly until harvest at 2 weeks, beginning immediately after the surgical procedure. Zebrafish were anesthetized, patted dry and placed on a balance. Weight was recorded as a percentage of initial weight ± SEM.

### Harvest procedure

At 2 weeks postoperatively, the SBS and sham zebrafish were anesthetized, the proximal S1 intestine resected and placed in RNALater for RNA extraction (Sigma Life Sciences, #R0901).

### RNA extraction

The RNA was extracted with the Qiagen RNeasy mini kit and and RNA concentration was determined with the Nanodrop 2000 system (Thermo Scientific). The three samples with the highest RNA concentration were selected to make cDNA libraries to be sequenced while the remaining samples were analyzed by RT-qPCR.

### RNA sequencing

After RNA extraction, the RNA integrity was determined with a bioanalyzer (Agilent Technologies) and found to have RNA integrity numbers (RIN) greater than 9.3. Multiple samples had RIN of 10.0, indicating high quality RNA. Ex-Fold External RNA Controls Consortium (ERCC) controls (Ambion, Foster City, CA) were added to the samples prior to cDNA library creation [8]. ERCC controls contain two mixes of 92 sequences not found in eukaryotes, at different concentrations. The libraries were initially sequenced to 10 million reads and a power analysis completed with the Scotty algorithm [9]. The libraries were then deep sequenced further to get a total sequence of 50 million reads. The RNA short reads were sequenced and all samples were evaluated for quality with the FastQC bioanalyzer [10]. Sequences with low Phred quality scores were removed with Trimmomatic [11]. The remaining RNA sequences were aligned to the *Danio rerio* reference genome generated by the Wellcome Trust Sanger Institute (danRer7) [12] downloaded from UCSC genome database using RNA-star short read aligner and the ENCODE recommended parameters [13]. The read counts per transcript were found with the HTSeq-count python script [14]. Reads per kilobase per million mapped reads (RPKM) were produced with the edgeR [15] R/Bioconductor software package [16]. Differential gene expression was analyzed with the Remove Unwanted Variation R/Bioconductor software package (RUVSeq) [17] combined with edgeR. Analysis was performed with Ingenuity pathway analysis (http://www.ingenuity.com) and Gene ontology (GO) enrichment using the GOstats R/bioconductor software [18] and Gene Ontology Consortium (geneontology.org). Gene signatures for differentially expressed genes with false discovery rate (FDR)-corrected p-values <0.05 were converted with the NCBI homologene into corresponding human genes. The relative log expression graphs and principal component graphs were produced with the EDASeq R/Bioconductor software. The threshold for significant gene expression was expressed as log fold change ≥1.5 or ≤ −1.5.

### Reverse transcription quantitative polymerase chain reaction (RT-qPCR)

Seven-hundred-fifty nanograms of RNA as calculated after determination of the RNA concentration of the samples by the Nanodrop 2000 (Thermo Scientific) were reverse-transcribed to cDNA with iScript Reverse Transcription Supermix (Bio-Rad, #170-8841) from each sample. RT-qPCR was performed in triplicate according to the SYBR green protocol with SYBR-Green I Master Mix (Roche #4717516001) and the LightCycler 480 II (Roche) with the primers in Additional file 1: Table S1 and positive and no template negative controls, and melting curve analysis according to manufacturer instructions. Gene expression was normalized to a housekeeping gene, elf1-alpha (elf1a). Outliers were determined by ROUT method with a Q value of 0.5%. Statistical significance was determined by a student’s t-test in Prism Graphpad software. Significance was determined by *p*-value of <0.5.

### Immunofluorescence staining for β –catenin and counting

Proximal (S1) intestines were harvested from sham or SBS fish. An additional segment was harvested as well: the more distal (S4) segment of the intestine that remains after the creation of SBS, which was also harvested in each group (*n* = 5 for sham proximal, sham distal and SBS proximal, *n* = 6 for SBS distal). The intestinal samples were oriented, formalin fixed, paraffin embedded and cut onto slides. Slides were subjected to a deparaffinization and rehydration process. Antigen retrieval was performed in a decloaking chamber with slides immersed in a 0.1 M sodium citrate solution for 10 min at 120°. Once cooled, slides were washed in PBS with 0.1% Tween (PBSt) for 15 min and blocked with 10% normal goat serum in PBSt for 1 h. Primary antibody anti-β-catenin (1:1,000, C2206 rabbit polyclonal; Sigma) was applied and samples were incubated overnight at 4 °C. The next day, slides were washed with PBSt for 15 min and exposed to secondary antibody goat-anti-rabbit 488 (1:500; Molecular Probes) for 2 h at room temperature. Slides were rinsed, counterstained and mounted in Vectashield with DAPI (Vector). Imaging was performed with an immunofluorescent microscope (Leica DM5500). β –catenin was quantified in the epithelium by counting the number of β –catenin positive cells per hemivillus, defined as cells from the intervillus pocket to the tip of the villi. All areas with complete hemi-villus were included in the quantification. A qualified, blinded observer carried out the quantification. The measurements were recorded as the percentage of β –catenin positive cells per total DAPI-stained epithelial cells per hemi-villus ± SEM.

## Results

### After 2 weeks of SBS, zebrafish intestine has a high number of upregulated differentially expressed genes based on RNA sequencing

**Fig. 1:**
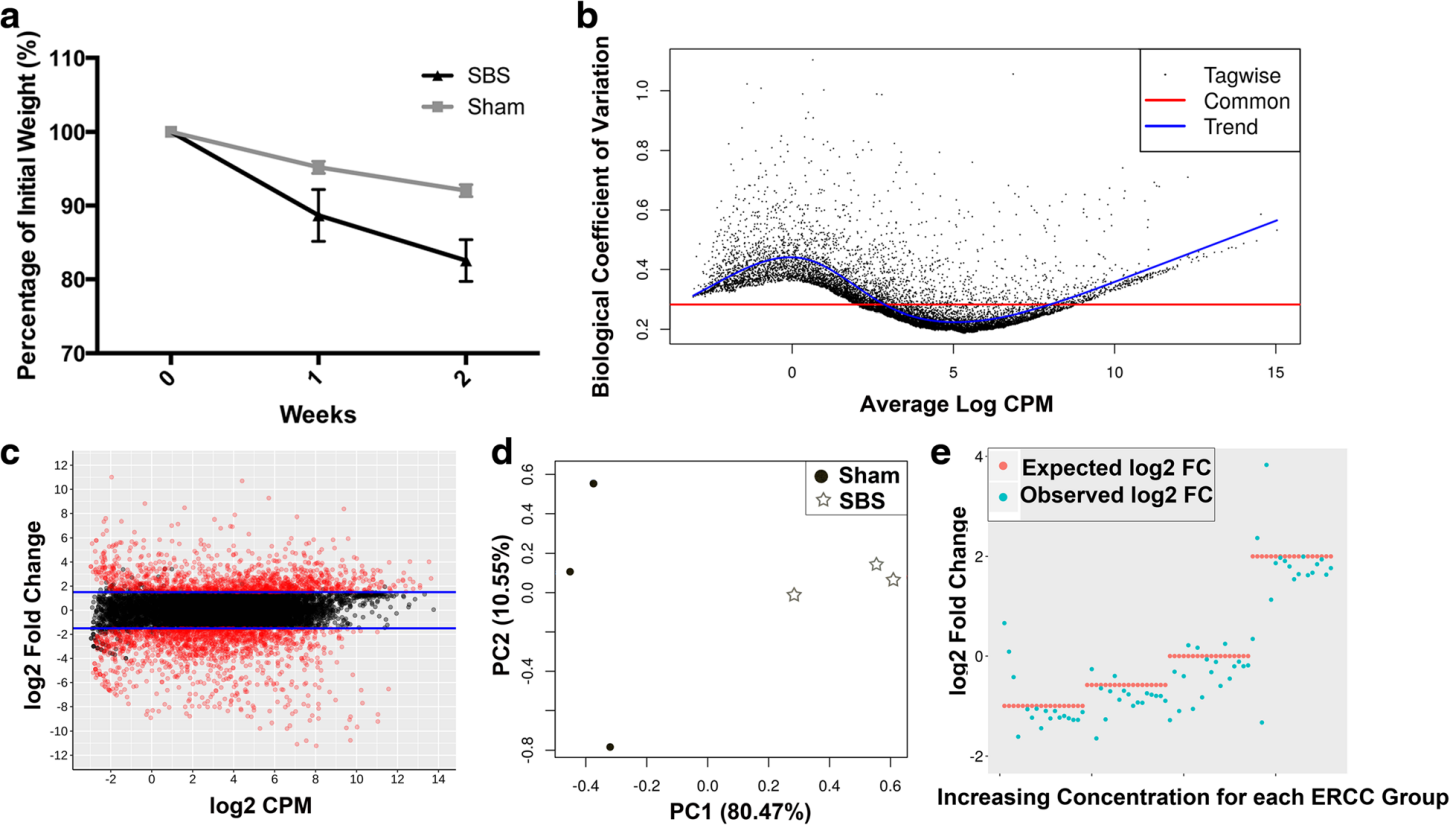
SBS zebrafish have significantly different gene expression than sham. **a** SBS zebrafish lost a significant percentage of preoperative weight compared to sham zebrafish over 2 weeks. **b** The biological coefficient variation over average log counts per million mapped reads with the trend *line in blue* and the common in *red*. **c** The volcano plot of sequenced genes showing log fold change. Each gene is expressed by a dot with the *red dots* representing an FDR corrected *p*-value <0.05 with significant upregulated SBS genes >1.5 and downregulated genes <1.5. **d** Principal component analysis compared the SBS zebrafish intestine to the sham group. (E) ERCC graph comparing observed log_2_ fold change (FC) to the expected log_2_ FC with good correlation

Correlating with multiple previous experiments in this model, and the human condition, the SBS zebrafish lost a significant percentage of initial weight when compared to the sham group (Fig. 1a) (82.5% vs 92.0%, *p* < 0.05). RNA-seq genome wide analysis was performed on three SBS and sham zebrafish proximal intestine (Table 1). The SBS group had 1346 significant differentially expressed genes that were upregulated while there were 678 genes that were downregulated (Table 2).

**Table 1:** The number of sequenced reads mapped to the SBS and sham zebrafish intestine

Sample ID	Raw reads	Raw combined	Clean reads (percentage)	Mapped reads (percentage)
Sham 1	4.80E + 07	6.70E + 07	65982094 (98.06%)	63078706 (95.60%)
Sham 2	4.40E + 07	6.20E + 07	60497171 (97.63%)	56858850 (93.99%)
Sham 3	4.80E + 07	6.80E + 07	67161466 (98.13%)	64522681 (96.07%)
SBS 1	1.90E + 07	3.70E + 07	36112322 (96.71)	33326196 (92.28%)
SBS 2	4.70E + 07	6.60E + 07	65338859 (98.58%)	61739067 (94.49%)
SBS 3	4.60E + 07	6.50E + 07	63391449 (97.98%)	59498306 (93.86%)

**Table 2:** The number of differentially expressed genes between SBS and sham zebrafish intestine

	Number of DEGs	Number of up-regulated DEGs	Number of down-regulated DEGs
SBS vs Sham	2024	1346	678

### SBS zebrafish intestine had increased gene expression differences when compared to the sham group based on RNA-seq analysis

RNA sequencing of SBS and sham zebrafish demonstrated low variation between all samples as shown in the biological coefficient of variation graph (Fig. 1b) with many significantly upregulated and downregulated SBS genes with a ≥1.5 or ≤ −1.5 cutoff (Fig. 1c). Principal component analysis showed marked differences in the SBS zebrafish intestine as compared to the sham zebrafish, more so than the differences between samples (Fig. 1d). External controls were added to the samples prior to sequencing to evaluate sequencing quality. The ERCC graph shows that the observed log_2_ fold change (FC) was very close to the expected log_2_ FC, confirming high quality sequencing of the samples (Fig. 1e).

### RNA sequencing data was of high quality, including the ERCC controls

Sequencing reads aligned to the zebrafish genome were used to generate a number of data quality assessment plots. After data processing, a ‘smear’ plot (Fig. 1c) was produced of the determined log_2_ fold-change (logFC) versus the average log_2_ counts per million mapped reads (logCPM). Each point belongs to a specific genetic element for which an Ensembl gene ID exists in the DanRer 7 annotated genome. Genetic elements colored red correspond to elements that have been identified to be statistically significant (FDR corrected *p*-value < 0.05). No artifacts were detected at any of the lower or higher ranges of the log_2_CPM.

The External RNA controls consortium ExFold probes (ERCC) were included as a ‘spike-in’ control to assess the performance of the statistical analysis. The ERCC probes consist of two mixes of 92 sequences not found in Eukaryotes. Each mix contains the same 92 sequences but at different concentrations, which span six log decades. The measurements for these sequences was used to construct an expected versus observed log_2_FC plot (Fig. 1e) which demonstrates excellent agreement considering the potential for bias introduced by recombinant reverse transcription used to generate the cDNA library.

The R/Bioconductor software edgeR also facilitated an assessment of the biological coefficient of variation (BCV) across all of the genes measured [19]. A graph of the calculated BCV versus the log_2_CPM (Fig. 1b) shows an average BCV of 28% and the vast majority of measurements under 40% for all ranges of log_2_CPM.

The quality of the raw data was assessed by principle component analysis using the software R/Bioconductor software EDASeq [20]. Principle component analysis shows that the data is well separated on principle component axis 1 (80%) and fairly well on axis 2.

Gene set enrichment was performed using the R/Bioconductor software GOstats [18]. The subset of genes with FDR corrected *p*-values less than 0.05 were compared to all of the genes for which there was a measurement. GOstats performs hypergeometric testing comparing the distribution of gene ontology (GO) terms between the subset and the whole. If the ratio of genes corresponding a particular GO term is different between the subset and the whole, that GO term is overrepresented if the ratio of genes corresponding to the GO term is higher than the whole, and underrepresented if the ratio of genes corresponding to the GO term is lower than the whole.

### SBS results in increased gene expression associated with proliferation, inflammation and immune system activation

**Fig. 2:**
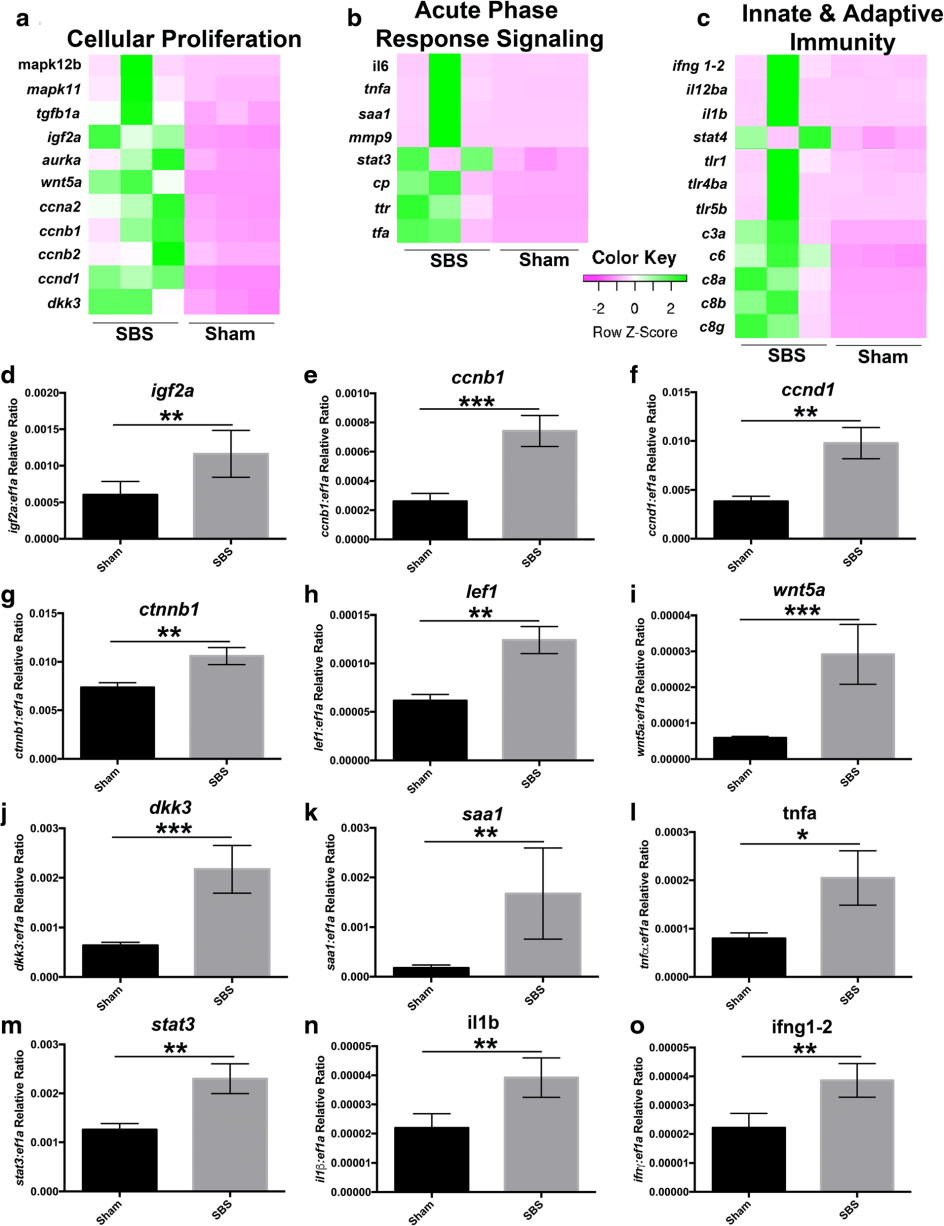
SBS zebrafish have increased expression of proliferation, inflammation and immunity. **a**-**c** Heat-maps of RNA-seq analysis show the gene expression differences between SBS and sham zebrafish in regards to cellular proliferation, acute phase response signaling and innate and adaptive immunity. RT-qPCR confirmation was performed on examples of cellular proliferation [*igf2a* (**d**), *ccnb1* (cyclin B1, (**e**)), *ccnd1* (cyclin D1, (**f**)), *ctnnb1* (β-catenin, (**g**)), *lef1* (**h**), *wnt5a* (**i**), *dkk3* (**j**)], acute phase response signaling [*saa1* (**k**), *tnfa* (**l**), *stat3* (**m**)] and innate and adaptive immunity (*il1b* (**n**), *ifng1-2* (**o**)]. ^*^ *denotes p < 0.5,* ^**^ *p < 0.1,* ^***^ *p < 0.001*

RNA-seq analysis heat-maps revealed many significant differences. SBS zebrafish intestine demonstrated upregulation of genes associated with cell proliferation, acute phase response signaling and innate and adaptive immunity (Fig. 2a–c). Key genes in cell proliferation were confirmed with RT-qPCR showing a significant increase in *igf2a, ccnb1 (cyclin B1) and ccnd1 (cyclin D1)* (Fig. 2d–f). Another signaling pathway involved in cell proliferation is the Wnt pathway, which is significantly increased with expression of *ctnnb1 (β-catenin), lef1, wnt5a and dkk3* (Fig. 2g–j). This correlated with an increased expression of β –catenin detected by immunofluorescence in the proximal intestine of SBS fish compared to the proximal segment in Sham fish (4.676% +/− 0.4711, *n* = 39 vs. 2.969% +/− 0.4128 *n* = 27 *p* =0.0123). Furthermore, β –catenin-positive cells were detected in greater numbers in SBS proximal bowel compared to SBS distal bowel (4.676% +/− 0.4711 *n* = 39 vs 2.379% +/− 0.4782, *n* = 47 *p* = 0.0011). No significant difference was noted between Sham proximal and distal intestine (Additional file 2: Figure S1).

Acute phase response signaling is also increased in SBS intestine when compared to the sham group, confirmed with significantly increased expression of *saa1, tnfa* and *stat3* on RT-qPCR (Fig. 2k–m). Innate and adaptive immunity is also significantly increased within the SBS group, confirmed by *il1b (il-1β)* and *ifng1-2* (*ifn-γ)* RT-qPCR (Fig. 2n–o).

### SBS results in increased gene expression in the intestine of genes commonly expressed in the liver and also those associated with barrier function

**Fig. 3:**
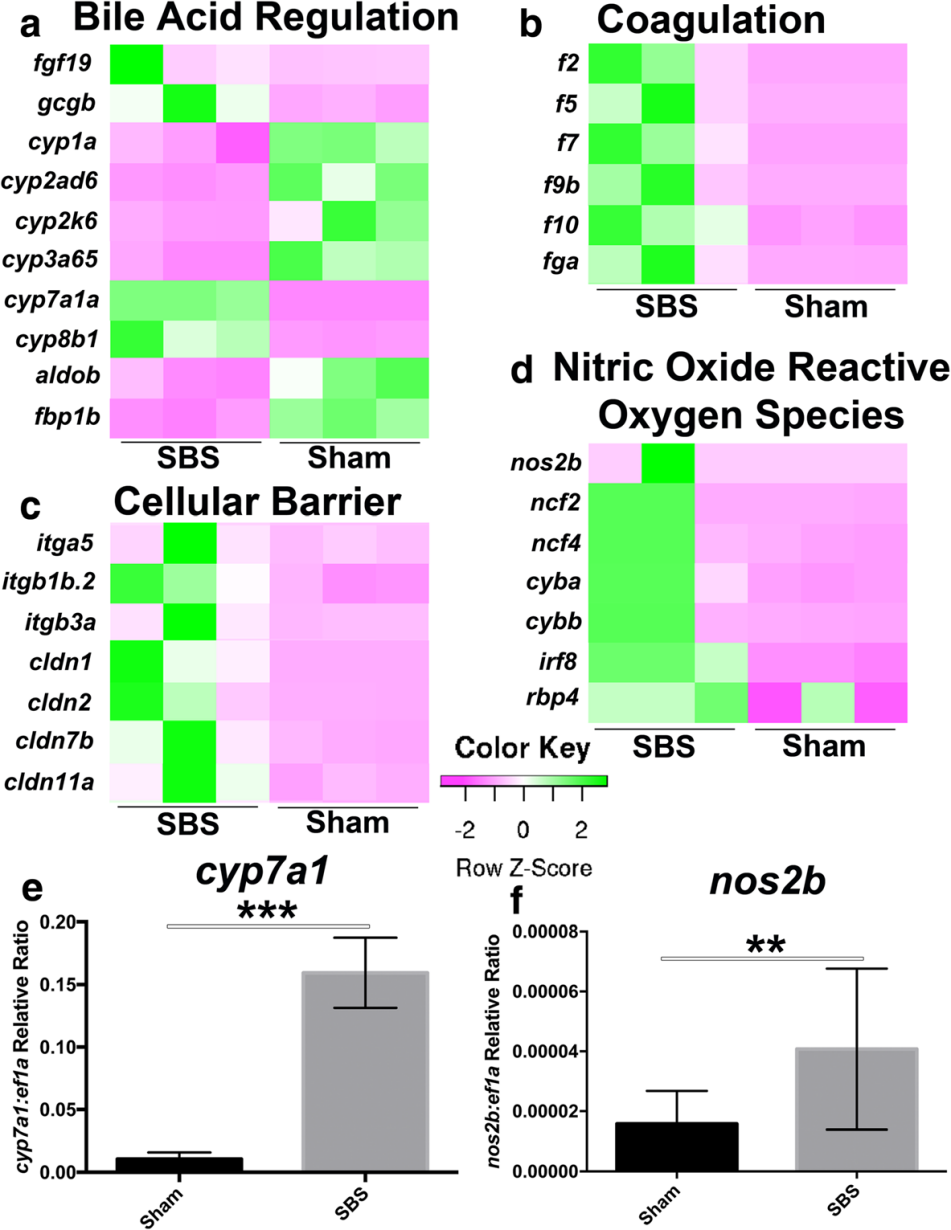
SBS zebrafish have widespread increased systemic gene expression compared to sham. **a**–**d** Heat-maps of RNA-seq analysis show the differences in gene expression between the SBS and sham zebrafish broken down into bile acid regulation, nitric oxide reactive species production, cellular barrier and coagulation. RT-qPCR confirmation was performed on bile acid reguation [*cyp7a1a* (**e**)] and nitric oxide reactive species production [*nos2b* (**f**)]. ^*^ *denotes p < 0.5,* ^**^ *p < 0.1,* ^***^ *p < 0.001*

**Fig. 4:**
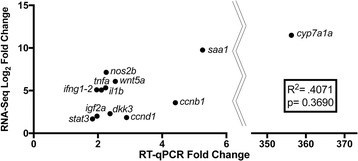
RT-qPCR confirms results of RNA-seq analysis. The ratio of the log_2_ fold change (FC) from the RNA-seq analysis was compared to the fold change seen with RT-qPCR of a subset of genes. No significant difference was found between the RNA-seq analysis and the confirmatory RT-qPCR

Heat-maps of RNA-seq analysis revealed increased expression in SBS of genes more usually identified in liver in the condition of bile acid regulation and hepatic fibrosis, as well as genes that regulate nitric oxide production, the cellular barrier and coagulation pathways (Fig. 3a–d). Expression in the SBS intestine of genes usually identified in cholestatic, hepatic fibrosis, bile acid regulation and coagulation processes in the liver was confirmed by RT-qPCR, with the identification of significantly elevated *cyp7a1a* (cytochrome p450 7A1) (Fig. 3e). Nitric oxide reactive species were also increased in SBS zebrafish, confirmed by *nos2b* (nitric oxide synthase 2) on RT-qPCR (Fig. 3f). The log_2_ fold change (FC) from the RNA-seq analysis of the SBS vs sham was compared to the RT-qPCR FC with no significant difference between the results from the RNA-seq analysis and the confirmatory RT-qPCR (Fig. 4).

### SBS over- and under-represents multiple disparate biological processes

**Fig. 5:**
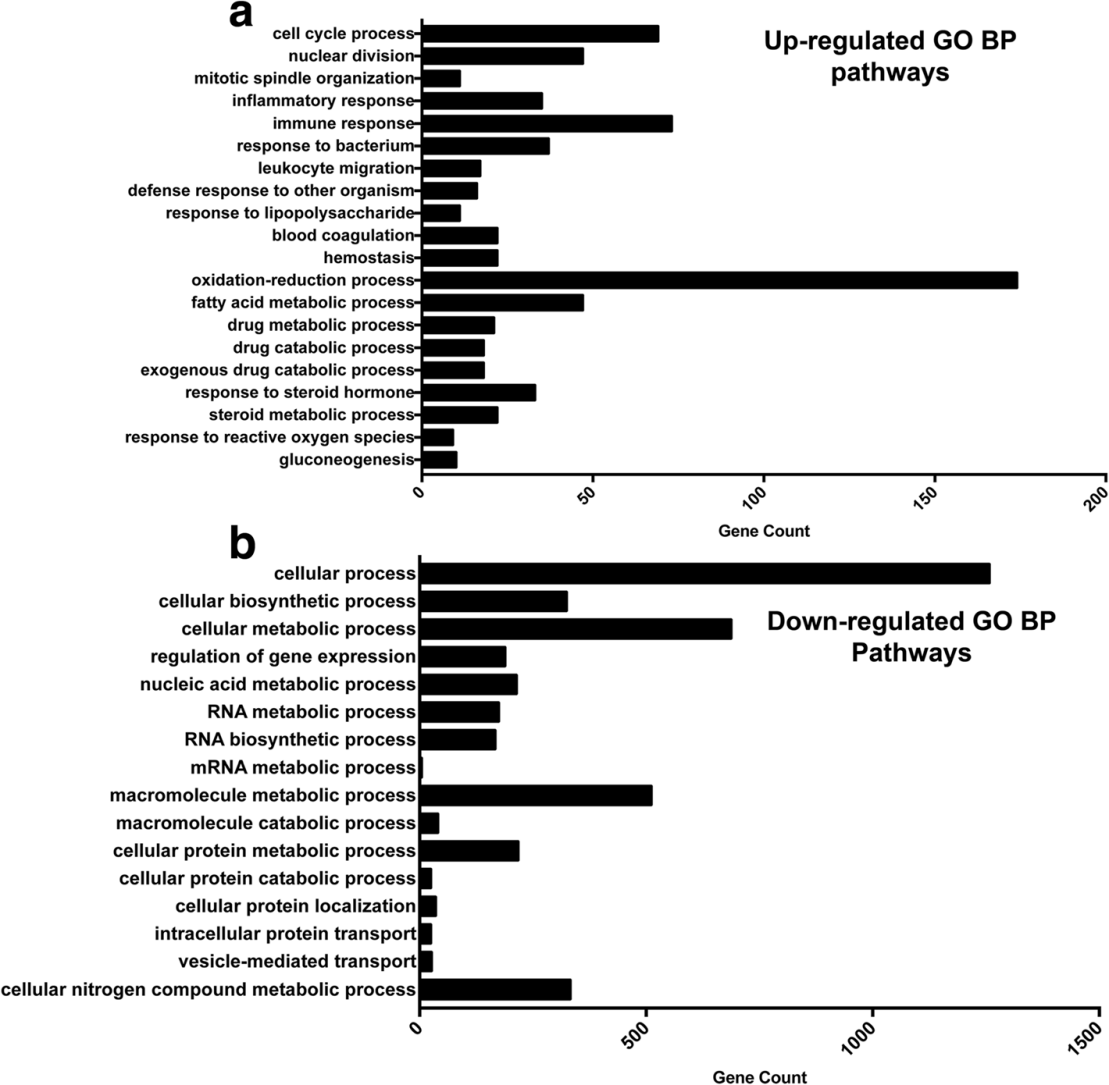
SBS positively and negatively alters multiple biological processes. GO analysis demonstrated multiple systemic processes that are either upregulated (**a**) or downregulated (**b**) in SBS as compared to sham zebrafish

**Fig. 6:**
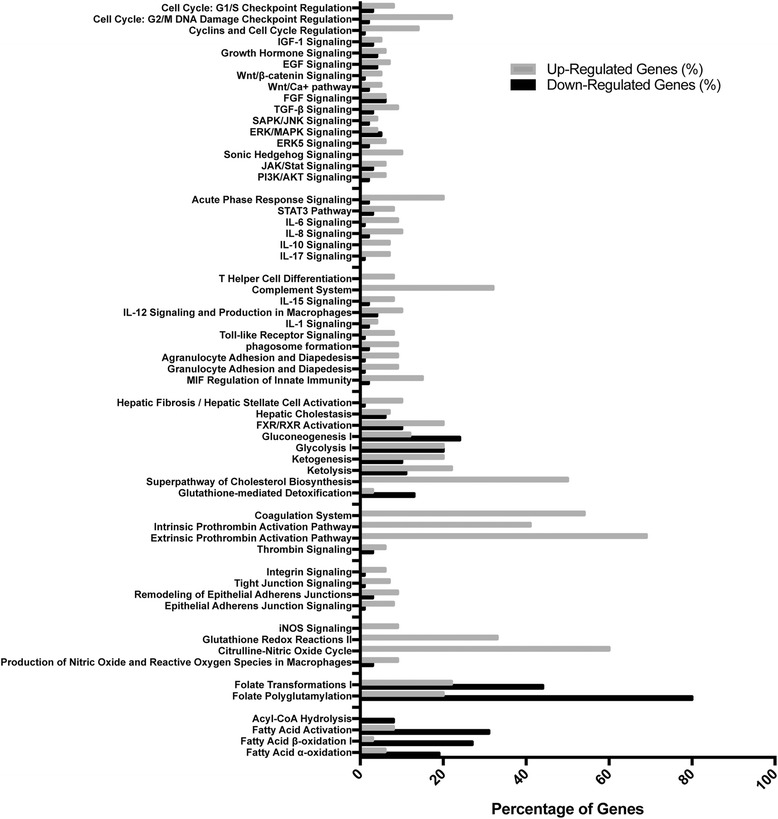
SBS leads to many significant alterations in systemic pathways within zebrafish intestine as compared to sham. Ingenuity pathway analysis (IPA) reveals many pathways with upregulated genes [12] and downregulated genes [31] expressed as a percentage of genes

Significantly different genes were then compared to the Danio rerio genome in the Gene Ontology Consortium database to determine which processes were either over- or under-represented. GO analysis of over-represented (Fig. 5a) and under-represented (Fig. 5b) processes are shown with the associated gene count. The complete list of over-represented processes and under-represented processes are located in Additional file 3: Table S2 and Additional file 4: Table S3. The significantly different genes were also analyzed with Ingenuity pathway analysis and were graphed, showing the percentage of genes upregulated or downregulated in canonical pathways (Fig. 6). The complete list of percentage of genes that are upregulated or downregulated with the pathways are shown in Additional file 5: Table S4.

## Discussion

### In-depth analysis of changes in the transcriptome have not been possible in alternative models of adaptation

After SBS has been established following massive small bowel resection, the degree of adaptation that may occur in the residual intestine is highly variable and sometimes fails to occur at all. When adaptation is inadequate, salvage therapies including intestinal transplant are still possible, but costs are high, supplies of donor organs are low, and lifelong immunosuppression is required [21, 22]. Because SBS and subsequent adaptation occur in a complex in vivo milieu, it is impossible to dissect the critical mechanisms in reductionist in vitro models.

Adaptation models have been reported in mice and larger mammals [23]. As in the human condition, these employ massive small bowel resection and are complicated, time consuming and associated with low survival and high cost. For these reasons, adaptation is still poorly understood, limiting human therapies. We recently developed a novel zebrafish model for SBS in order to identify cellular and molecular mechanisms that are critical to ensure and promote intestinal adaptation, and to develop and confirm human regenerative medicine targets [7]. Our initial data indicated that SBS resulting from a proximal intestinal diversion results in an increase in BrdU+ cells in the intervillus pocket at 2 weeks that resolves by 4 weeks, with an increase in epithelial surface area at that point. We therefore chose to investigate the 2-week timepoint.

In matched samples of small intestine from SBS zebrafish compared to sham-operated fish, we found 1346 upregulated genes and 678 downregulated genes, of which a subset were confirmed by PCR. The SBS fish lost weight due to their severely truncated intestinal length, as in the initial description of this model. The upregulated genes were associated with cell proliferation, acute phase response signaling, innate & adaptive immunity, production of nitric oxide & reactive oxygen species, and the cellular barrier. Other upregulated genes include those related to hepatic fibrosis, cholestasis, bile acid regulation or coagulation although they were expressed in the intestine. The downregulated genes were related to folate synthesis, gluconeogenesis, glycogenolysis, fatty-acid oxidation and activation and drug and steroid metabolism. We assessed key genes identified by RNA-Seq with RT-qPCR, which confirmed the RNA-Seq data and employed ERCC controls. The samples have very low variance between replicates and a fairly large difference in variance between the groups. The average coefficient of variation for 300 series, 400 series and between groups using RPKM (Additional file 6: Table S5) is 45, 52 and 92% respectively amongst the 14,797 genes detected. Power analysis of a small subset of the data (2 + 2 and 10 million reads SE) was performed which indicated the criteria (depth of sequencing) whereby three replicates would have sufficient statistical power (α = 0.05, β = 0.2) as calculated by the Scotty web-based algorithm [9].

### Expansion of the epithelium is the *condicio sine qua non* of adaptation, and gene expression related to cellular proliferation was increased in the SBS group

Intestinal adaptation is a critical physiological response necessary to increase intestinal surface area to compensate for the loss of intestine. We, and others have consistently measured increases in villus height and crypt depth in the adaptive state [7, 24]. Individual growth factors have been investigated in SBS models, with conflicting results. For example, Epidermal growth factor (EGF), glucagon-like peptide 2 (Glp2), growth hormone and insulin-like growth factor (IGF) are factors that have been confirmed to stimulate adaptation in multiple rat, rabbit and piglet studies [25–32]. However, studying the addition or subtraction of each of these factors has yielded variable results in animal models, e.g. although both EGF and IGF signaling have been shown to be important, adaptation still occurs with disruption of both EGFR and IGF1R [33]. However, taken together, SBS intestine at 2 weeks demonstrates upregulation of multiple key proliferative genes. Some of these genes are proposed to relate to cell cycle regulation such as *ccnb1* (cyclin B1,3.6 log_2_ FC) and *cdk1* (cyclin-dependent kinase 1,3.7 log_2_ FC) or Wnt signaling, such as *ccnd1 (*cyclin D1,1.8 log_2_ FC) and *wnt5a* (6.1 log_2_ FC). We also detected an increase in cells that stained for the β –catenin protein by immuofluorescent staining in the SBS fish, as compared to sham. This observation was strengthened by also noting an increase in the β –catenin-positive cell number in the proximal SBS segment compared to the distal segment retained in the fish after the SBS surgery, an increase that was not identified in sham controls. Interestingly, *dkk3* (2.3 log_2_ FC), a secreted Wnt antagonist, was also increased. In a study of enteral atrophy followed by refeeding and therefore epithelial expansion in the mouse, *wnt5A*, *cyclin D1* and *c-Myc* all decline without enteral nutrition and then are restored with the reintroduction of enteral nutrition and the subsequent adaptive response [34].

Other genes associated with proliferation such as *tgfβ* (1.6 log_2_ FC) have multiple roles - although TGFβhas a significant role of modulating the intestinal epithelium, particularly after injury [35], has been shown to inhibit cyclin D1 expression in vitro in rat intestinal epithelial cell lines [36] and is an immunosuppressive cytokine that inhibits intestinal T cell activation [37]. As in this zebrafish model, *tgfβ* has been noted to rise with refeeding in the mouse model noted above [34].

### Signaling that is associated with innate and adaptive immunity in SBS is increased

Expression of *stat4* (1.8 log_2_ FC), *il12b* (4.8 log_2_ FC) and *ifng1-2* (*ifnγ*, 5.1 log_2_ FC) all increased, as did *tlr1* (2.6 log_2_ FC), *tlr5* (3.1 log_2_ FC) and *tlr4* (3.8 log_2_ FC). There are 2 types of T cells; Type 1 helper (Th1) cells produce IFNγ, IL2 and TNFβ which activate macrophages and are responsible for cell-mediated immunity as well as phagocytic dependent responses. Type 2 helper cells (Th2) cells are responsible for antibody production [38]. In human intestine, T cells associate within Peyer’s patches and active stat4 is a transcription factor that is an essential component of IL-12 mediated T helper-1 cell differentiation expressed in that location, inducing IFNγ release and increased natural killer cell cytotoxicity [39]. Activation by microbes cause an elevation in IL12, 15, 18 and therefore the release of IFNγ. Exogenous administration of both IL12 and IL15 leads to lesions of the GI tract, increased acute phase reactants and pro-inflammatory cytokines, and NK Cell apoptosis [40]. IL12 also has the ability activate the *stat4* pathway, and can activate *stat3* (1.68 log_2_ FC) as well as *stat1b* (2.16 log_2_ FC) [41]. Toll-like receptor 4, TLR4, the receptor for Lipopolysaccharide or LPS, has been shown to be elevated in both epithelial atrophy [42] in a mouse model and adaptation in a rat model [43].

### Many components of the transcriptome associated with acute phase response signaling and complement system activation are elevated

Elevations were noted in *il6* (8.7 log_2_ FC), *il6r* (IL6 receptor, 3.6 log_2_ FC), *saa1* (9.7 log_2_ FC), *stat3* (1.6 log_2_ FC), *tnfa* (5.1 log_2_ FC) and *il1b* (5.3 log_2_ FC). In rats, SBS is a proinflammatory state that is magnified by sepsis [4]. IL6 is a cytokine that is associated intimately with inflammation and acute phase response signaling. Early after induction of inflammation, IL6 is elevated and is required for efficient stimulation of epithelial cell proliferation after intestinal injury within a mouse model [44]. SAA1 is highly conserved and found to play a role in lipid metabolism as well as bacterial clearance and has a possible role in regulation of inflammation by extending the lifespan of neutrophils and activating proinflammatory cytokines (IL6, IL8, IL1beta, CXCL1, CXCL2) through several receptors including TLR2 and TLR4 [45].

Both IL22 and elements of the complement system are elevated: *c3a.1* (8.5 log_2_ FC), *c3b.1* (8.3 log_2_ FC), *il22* (6.4 log_2_ FC), *il1b* (5.3 log_2_ FC) Activation of complement leads to non-specific immunity and inflammation secondary to active by-products. The main components are the C3 and C4 complement with the addition of other factors resulting in a membrane attack complex that attacks the bacterial cell wall. IL22 has been shown to play a critical protective role by increasing the expression and bacterial binding of complement C3 after systemic bacterial translocation of potentially virulent species. In addition to IL22 induction, C3a and C5a are also known to recruit IL1β producing inflammatory cells [46]. This inflammatory milieu may underpin other observations such as elevations of *nos2b* (7.1 log_2_ FC).

### Genes in the bile acid biosynthesis pathway are strongly upregulated, with an elevation of *cyp7a1a*, which is more usually identified in the liver

Hepatic cholestasis, cirrhosis and even liver failure are often associated with SBS, and the main culprit for these changes is usually identified as the intravenous feeding that supports patients who do not gain enough intestinal adaptation for enteral autonomy [47]. In brief, bile acids are conserved in the intestine via enterohepatic recycling, and act on the nuclear farnesoid X receptor (FXR) receptor to activate FGF19 expression. FGF19, encoded by *fgf1*9 (5.6 log_2_ FC) is atypical in that it acts as a hormone, and after portal circulation to the liver represses transcription of *cyp7a1a* (11 log_2_ FC), which encodes cytochrome P450 family 7 subfamily A member 1, also known as cholesterol 7-α-hydroxylase, the rate limiting enzyme in bile acid synthesis from cholesterol [48]. *FGF19* transgenic mice remain lean on an obesogenic diet and have an increased metabolic rate [49], neither of which is desirable in SBS patients who struggle to maintain or gain weight. This, however, is just a sketch of the effects within this pathway, and future work is required to fully understand these identified changes in the transcriptome in SBS.

Additionally, *fgf21* (3.2 log_2_ FC) was increased in SBS zebrafish intestine. In pediatric intestinal failure, an increase in FGF21 in the serum is significantly associated with hepatic steatosis, and correlated with the duration of IV nutritional support. Liver steatosis was coupled with the progression of fibrosis without accompanying inflammation [50]. The zebrafish in this model do not receive IV nutritional support and therefore may be useful to further define what changes derive from SBS alone.

### Genes associated with maintenance of the intestinal epithelial barrier were markedly changed

Expression of *cldn1* (Claudin 1, 7.4 log_2_ FC), *cldn2* (7.0 log_2_ FC), *cldn7b* (2.4 log_2_ FC) and *cldn11a* (2.3 log_2_ FC) were all increased, as were several of the integrins. The isolated SBS barrier function was found to be relatively unchanged in regards to claudin-1, claudin-2 and claudin-4 with a decrease in occludin expression when compared to sham group [51]. Other treatments can decrease the level of barrier function. Enteral nutrition deprivation causes a decrease in occludin, ZO-1 and claudin-4 with a marked increase in FITC-dextran permeation [42].

We chose to investigate a 2-week time point because it corresponded to an observation of statistically significantly increased BrdU-positive cells in the intestine of fish with SBS, and our long-term goal is to understand the cellular and molecular mechanisms of intestinal adaptation. However, in order to dissect these results reported above, it will be necessary to investigate other time points and to begin to assign gene expression signals to particular cellular compartments or cell types in future work. Additionally, the changes in the transcriptome related to immune and barrier functions immediately suggest a contribution of the microbiome, the diversity and alteration of which must be investigated.

## Conclusions

Short bowel syndrome is a highly complex disease process that is likely to have systemic effects including immune system activation, inflammation, changes to the coagulation and bile acid pathways, as well as altering the cellular barrier. Genes associated with proliferation and Wnt signaling are also notably increased. Given these widespread effects, further evaluation of SBS in vivo and the changes that SBS causes in the transcriptome may assist the discovery and translation of future human therapies.

## Additional files

Additional file 1: Table S1. List of RT-qPCR primers. (TIF 6990 kb)
Additional file 2: Figure S1. Increased β –catenin is detected by immunofluorescence in proximal SBS intestine compared to distal SBS and Sham intestine. Immunofluorescent detection of β –catenin identified more positive cells/hemivillus in SBS intestine compared to both the distal limb and sham proximal controls (A–C). Increased β –catenin is noted in the proximal SBS intestine (C; E) compared to distal SBS (D; E *p* = 0.001) and Sham proximal and SBS proximal intestine (A–C; E *p* = 0.012). No significant difference in β –catenin is seen between Sham proximal and distal intestine (A–B, E). A–D Scale 50 μm. (TIF 2151 kb)
Additional file 3: Table S2. GO enrichment analysis for biological processes over-represented by genes of SBS zebrafish intestine as compared to sham. (PDF 143 kb)
Additional file 4: Table S3. GO enrichment analysis for biological processes under-represented by genes of SBS zebrafish intestine as compared to sham. (PDF 135 kb)
Additional file 5: Table S4. IPA analysis of percentage of up- and downregulated genes within each significant pathway. (PDF 295 kb)
Additional file 6: Table S5. Results of the differential gene analysis with RPKM values and annotation for every gene measured. (XLSX 4103 kb)

## Abbreviations

BrdU: Bromodeoxyuridine
*c3a.1*: Complement component c3a, duplicate 1
*c3b.1*: Complement component c3b, duplicate 1
*ccnb1*: Cyclin B1
*ccnd1*: Cyclin D1
*cdk1*: Cyclin-dependent kinase 1
*cldn-1*: Claudin 1
*cldn11*: Claudin 11
*cldn-2*: Claudin 2
*cldn7b*: Claudin 7b
*ctnnb1*: Beta-catenin
*cyp7a1a*: Cytochrome p450 7a1a
dkk3: Dickkopf wnt signaling pathway inhibitor 3
EGF: Epidermal growth factor
ERCC: External RNA controls consortium
FDR: False discovery rate
*fgf19*: Fibroblast growth factor 19
*fgf21*: Fibroblast growth factor 21
*FXR*: Farnesoid X receptor
Glp-2: Glucagon-like peptide 2
GO: Gene ontology
*ifng1-2*: Interferon gamma 1–2
IGF: Insulin-like growth factor
*igf2a*: Insulin-like growth factor 2a
*il12b*: Interleukin 12 beta
*il1b*: Interleukin 1 beta
*il22*: Interleukin 22
*il6*: Interleukin 6
*il6r*: Interleukin 6 receptor
IV: Intravenous
*lef1*: Lymphoid enhancer-binding factor 1
*nos2b*: Nitric oxide synthase 2b
RIN: RNA integrity number
RPKM: Reads per kilobase per million mapped reads
RT-qPCR: Reverse transcription quantitative polymerase chain reaction
*saa1*: Serum amyloid A1
SBS: Short Bowel Syndrome
*stat3*: Signal transducer and activator of transcription 3
*stat4*: Signal transducer and activator of transcription 4
*tgfb*: Transforming growth factor beta
tlr1: Toll-like receptor 1
tlr4: Toll-like receptor 4
tlr5: Toll-like receptor 5
*tnfa*: Tumor necrosis factor alpha
TPN: Total parenteral nutrition
*wnt5a*: Wnt family member 5a
