# Supplementary figures and images for: Short bowel syndrome results in increased gene expression associated with proliferation, inflammation, bile acid synthesis and immune system activation: RNA sequencing a zebrafish SBS model
Source: BMC Genomics. 2017 Jan 25;18:23. doi: 10.1186/s12864-016-3433-4 (PMC5264326; doi:10.1186/s12864-016-3433-4)

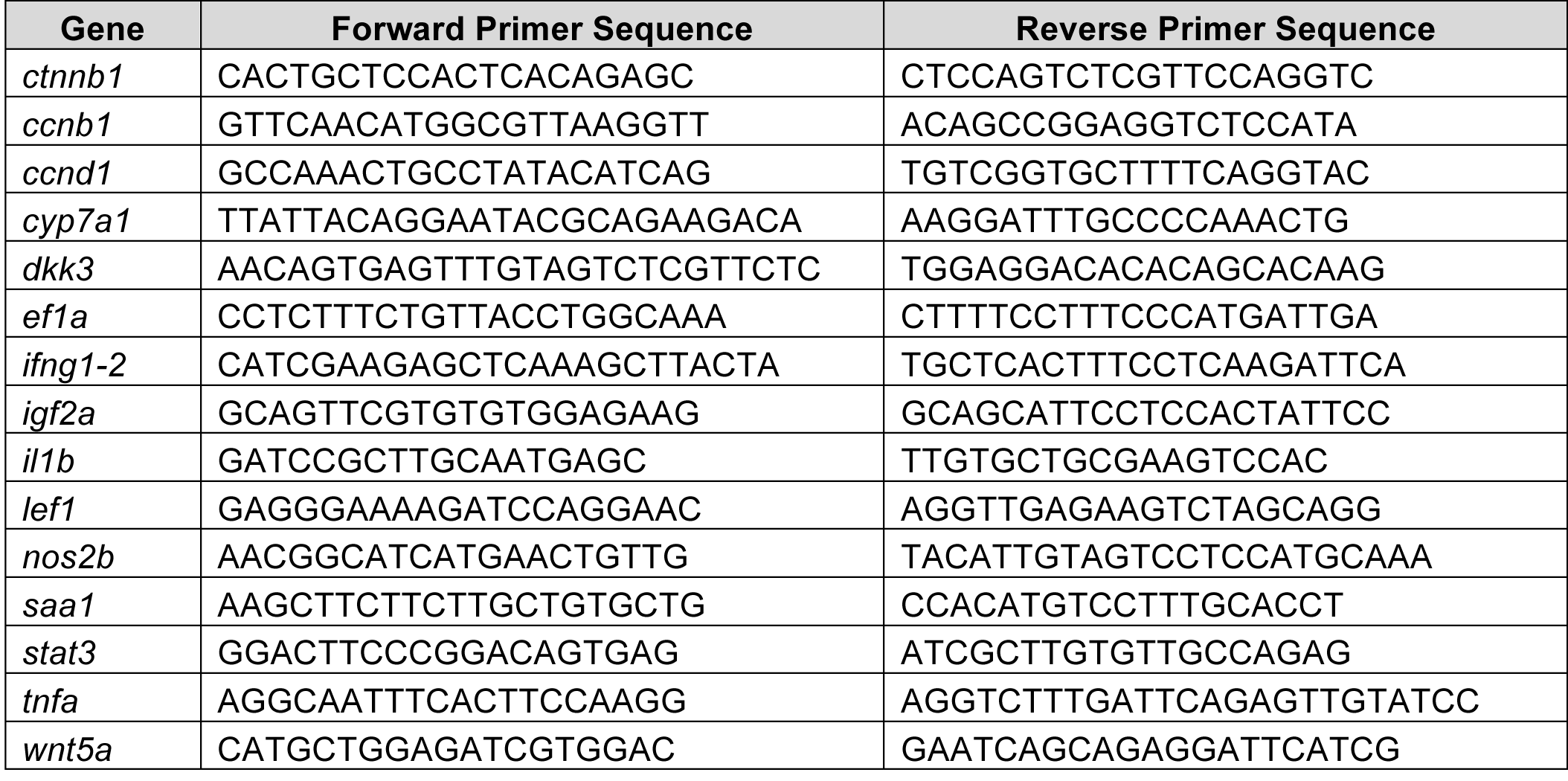

Supplement: Additional file 1: Table S1. — List of RT-qPCR primers. (TIF 6990 kb) [file 12864_2016_3433_MOESM1_ESM.tif]

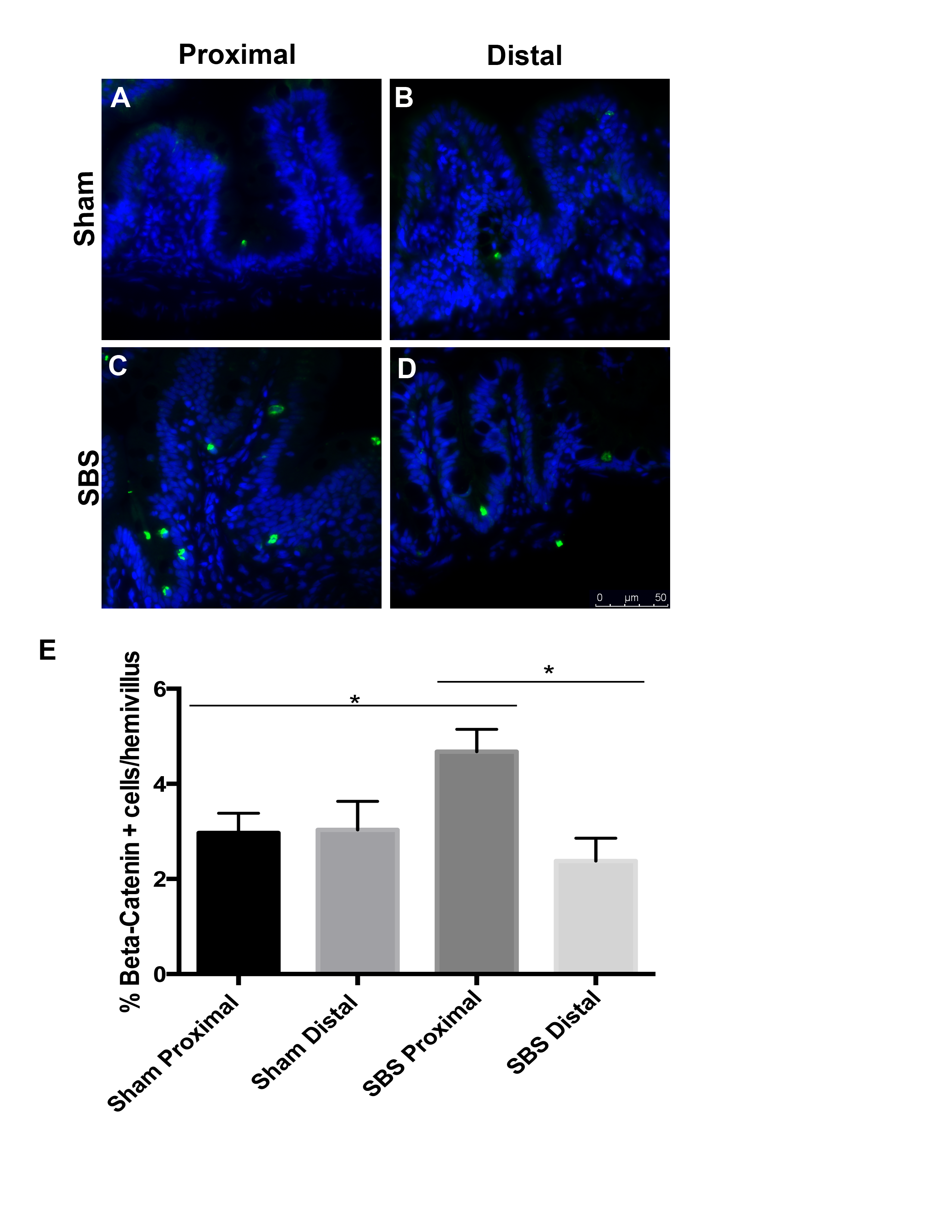

Supplement: Additional file 2: Figure S1. — Increased β –catenin is detected by immunofluorescence in proximal SBS intestine compared to distal SBS and Sham intestine. Immunofluorescent detection of β –catenin identified more positive cells/hemivillus in SBS intestine compared to both the distal limb and sham proximal controls (A–C). Increased β –catenin is noted in the proximal SBS intestine (C; E) compared to distal SBS (D; E p = 0.001) and Sham proximal and SBS proximal intestine (A–C; E p = 0.012). No significant difference in β –catenin is seen between Sham proximal and distal intestine (A–B, E). A–D Scale 50 μm. (TIF 2151 kb) [file 12864_2016_3433_MOESM2_ESM.tif]
