# Supplementary material for: Short bowel syndrome results in increased gene expression associated with proliferation, inflammation, bile acid synthesis and immune system activation: RNA sequencing a zebrafish SBS model
Source: BMC Genomics. 2017 Jan 25;18:23. doi: 10.1186/s12864-016-3433-4 (PMC5264326; doi:10.1186/s12864-016-3433-4)
Supplement: Additional file 3: Table S2. — GO enrichment analysis for biological processes over-represented by genes of SBS zebrafish intestine as compared to sham. (PDF 143 kb) [file 12864_2016_3433_MOESM3_ESM.pdf]

GOBPID	Pvalue	OddsRatio	ExpCount	Count	Size	Term
GO:0055114	1.04E-16	2.344469938	98.02425579	174	478	oxidation-reduction process
GO:0044699	6.27E-14	1.50725947	1164.191841	1301	5677	single-organism process
GO:0032787	1.36E-13	3.370204285	34.04189636	76	166	monocarboxylic acid metabolic process
GO:0006082	7.01E-12	2.208938451	76.49173098	132	373	organic acid metabolic process
GO:0019752	7.40E-12	2.296803628	68.28886439	121	333	carboxylic acid metabolic process
GO:0042221	1.35E-10	1.77259552	142.1146637	210	693	response to chemical
GO:0006631	1.58E-10	3.950910094	19.27673649	47	94	fatty acid metabolic process
GO:0009617	6.54E-10	4.700210571	13.94487321	37	68	response to bacterium
GO:0043436	1.05E-09	2.05631896	73.82579934	122	360	oxoacid metabolic process
GO:0006805	1.43E-09	9.014697877	6.767364939	23	33	xenobiotic metabolic process
GO:0017144	2.49E-09	10.28017945	5.94707828	21	29	drug metabolic process
GO:0009607	3.15E-09	3.094467716	25.22381477	54	123	response to biotic stimulus
GO:0006811	5.48E-09	1.863029648	93.92282249	145	458	ion transport
GO:0043207	7.49E-09	3.066272619	24.40352811	52	119	response to external biotic stimulus
GO:0051707	7.49E-09	3.066272619	24.40352811	52	119	response to other organism
GO:0050896	7.75E-09	1.384186198	497.9140022	596	2428	response to stimulus
GO:1901700	9.74E-09	2.991698949	25.22381477	53	123	response to oxygen-containing compound
GO:0009410	1.32E-08	4.1709423	13.94487321	35	68	response to xenobiotic stimulus
GO:0044710	1.50E-08	1.423235967	359.6957001	446	1754	single-organism metabolic process
GO:0071466	1.80E-08	4.490153173	12.30429989	32	60	cellular response to xenobiotic stimulus
GO:0009725	2.03E-08	2.652627528	31.17089305	61	152	response to hormone
GO:0044281	2.14E-08	1.650737816	142.3197354	201	694	small molecule metabolic process
GO:0033993	2.41E-08	3.182247239	21.12238148	46	103	response to lipid
GO:0014070	3.29E-08	2.806696975	26.65931643	54	130	response to organic cyclic compound
GO:0032870	3.87E-08	2.666236481	29.53031974	58	144	cellular response to hormone stimulus
GO:0044283	7.80E-08	2.618813605	29.32524807	57	143	small molecule biosynthetic process
GO:0042737	1.09E-07	8.79723127	5.331863286	18	26	drug catabolic process
GO:0042738	1.09E-07	8.79723127	5.331863286	18	26	exogenous drug catabolic process
GO:0051704	1.21E-07	2.37458194	36.09261301	66	176	multi-organism process
GO:0042493	2.44E-07	5.741385564	7.587651599	22	37	response to drug
GO:0006629	3.09E-07	1.807417046	78.95259096	120	385	lipid metabolic process
GO:0055085	3.15E-07	1.787777205	82.2337376	124	401	transmembrane transport
GO:0070887	4.57E-07	1.695598519	99.25468578	144	484	cellular response to chemical stimulus

GO:0006954	9.84E-07	3.196495699	15.99558986	35	78	inflammatory response
GO:0006955	1.90E-06	2.048034068	43.88533627	73	214	immune response
GO:0016042	2.37E-06	3.158276263	15.1753032	33	74	lipid catabolic process
GO:0009605	2.41E-06	1.733936789	77.72216097	115	379	response to external stimulus
GO:0006952	2.50E-06	2.242954451	32.81146637	58	160	defense response
GO:0007596	2.70E-06	4.530152912	8.407938258	22	41	blood coagulation
GO:0007599	2.70E-06	4.530152912	8.407938258	22	41	hemostasis
GO:0006820	2.82E-06	2.354770624	28.50496141	52	139	anion transport
GO:0050817	2.92E-06	4.286154963	9.023153252	23	44	coagulation
GO:0044282	3.68E-06	2.884398496	17.43109151	36	85	small molecule catabolic process
GO:0050878	4.81E-06	4.090760628	9.228224917	23	45	regulation of body fluid levels
GO:0072376	5.15E-06	35.05186386	2.050716648	9	10	protein activation cascade
GO:0071407	6.76E-06	2.640913481	19.89195149	39	97	cellular response to organic cyclic compound
GO:0048545	6.83E-06	2.941707718	15.79051819	33	77	response to steroid hormone
GO:0071383	7.47E-06	2.987600292	15.1753032	32	74	cellular response to steroid hormone stimulus
GO:0001676	8.92E-06	6.504968383	4.921719956	15	24	long-chain fatty acid metabolic process
GO:0006936	1.04E-05	4.640344921	7.177508269	19	35	muscle contraction
GO:0046364	1.18E-05	9.357142857	3.486218302	12	17	monosaccharide biosynthetic process
GO:0033559	1.43E-05	5.106598773	6.152149945	17	30	unsaturated fatty acid metabolic process
GO:0016053	1.82E-05	2.453983516	21.32745314	40	104	organic acid biosynthetic process
GO:0046394	1.82E-05	2.453983516	21.32745314	40	104	carboxylic acid biosynthetic process
GO:0019369	1.97E-05	7.242555495	4.101433297	13	20	arachidonic acid metabolic process
GO:0042742	2.23E-05	5.204627621	5.742006615	16	28	defense response to bacterium
GO:0071396	2.46E-05	2.695060208	16.61080485	33	81	cellular response to lipid
GO:0048878	2.54E-05	2.083642759	31.99117971	54	156	chemical homeostasis
GO:0009719	2.76E-05	1.723166929	61.52149945	91	300	response to endogenous stimulus
GO:0009611	2.94E-05	2.085390149	31.37596472	53	153	response to wounding
GO:0015711	3.05E-05	2.521381579	18.86659316	36	92	organic anion transport
GO:1901565	3.05E-05	2.521381579	18.86659316	36	92	organonitrogen compound catabolic process
GO:0044763	3.84E-05	1.230562094	943.5347299	1020	4601	single-organism cellular process
GO:0006690	4.02E-05	4.803604205	5.94707828	16	29	icosanoid metabolic process
GO:1901568	4.02E-05	4.803604205	5.94707828	16	29	fatty acid derivative metabolic process
GO:0019319	4.10E-05	8.572742023	3.281146637	11	16	hexose biosynthetic process
GO:0071391	4.19E-05	6.336356253	4.306504961	13	21	cellular response to estrogen stimulus

GO:0006812	4.23E-05	1.714174515	59.67585447	88	291	cation transport
GO:0006633	4.34E-05	3.330893768	10.25358324	23	50	fatty acid biosynthetic process
GO:0044242	4.34E-05	3.330893768	10.25358324	23	50	cellular lipid catabolic process
GO:0065008	4.47E-05	1.456739557	133.5016538	174	651	regulation of biological quality
GO:0003012	4.80E-05	3.906029332	7.792723264	19	38	muscle system process
GO:0000280	5.74E-05	2.122480679	27.47960309	47	134	nuclear division
GO:1901615	5.97E-05	2.45	18.6615215	35	91	organic hydroxy compound metabolic process
GO:0034220	7.09E-05	1.803739	45.73098126	70	223	ion transmembrane transport
GO:0010033	7.46E-05	1.521529871	96.99889746	131	473	response to organic substance
GO:0007186	7.47E-05	1.739218931	52.29327453	78	255	G-protein coupled receptor signaling pathway
GO:0000302	7.57E-05	11.68071313	2.460859978	9	12	response to reactive oxygen species
GO:0030261	9.32E-05	15.56803456	2.050716648	8	10	chromosome condensation
GO:0043627	9.92E-05	4.963360583	5.126791621	14	25	response to estrogen
GO:0044712	0.000114733	1.709502432	52.90848953	78	258	single-organism catabolic process
GO:0007059	0.000120336	2.932189542	11.48401323	24	56	chromosome segregation
GO:0006094	0.000139376	7.789189189	3.076074972	10	15	gluconeogenesis
GO:0072330	0.000151174	2.792915531	12.30429989	25	60	monocarboxylic acid biosynthetic process
GO:0030001	0.000168065	1.796617551	41.21940463	63	201	metal ion transport
GO:0071495	0.000170477	1.664260118	55.9845645	81	273	cellular response to endogenous stimulus
GO:0003008	0.000175818	1.705552609	50.24255788	74	245	system process
GO:0000226	0.000184019	2.153444555	22.55788313	39	110	microtubule cytoskeleton organization
GO:0098813	0.000188921	3.685536866	7.177508269	17	35	nuclear chromosome segregation
GO:0098542	0.000204163	2.963903793	10.45865491	22	51	defense response to other organism
GO:0031102	0.00024449	5.195526696	4.306504961	12	21	neuron projection regeneration
GO:0048285	0.000247913	1.923743788	30.55567806	49	149	organelle fission
GO:0032101	0.000257864	2.808485302	11.27894157	23	55	regulation of response to external stimulus
GO:0002237	0.00027625	4.606339519	4.921719956	13	24	response to molecule of bacterial origin
GO:0006006	0.00027625	4.606339519	4.921719956	13	24	glucose metabolic process
GO:0015698	0.000276677	3.345276873	7.997794928	18	39	inorganic anion transport
GO:0016054	0.000353717	2.72300028	11.48401323	23	56	organic acid catabolic process
GO:0046395	0.000353717	2.72300028	11.48401323	23	56	carboxylic acid catabolic process
GO:0007052	0.00038367	5.355732829	3.896361632	11	19	mitotic spindle organization
GO:0032496	0.00038367	5.355732829	3.896361632	11	19	response to lipopolysaccharide
GO:0006584	0.000426607	23.33009709	1.435501654	6	7	catecholamine metabolic process

GO:0007076	0.000426607	23.33009709	1.435501654	6	7	mitotic chromosome condensation
GO:0009712	0.000426607	23.33009709	1.435501654	6	7	catechol-containing compound metabolic process
GO:0010872	0.000426607	23.33009709	1.435501654	6	7	regulation of cholesterol esterification
GO:0010873	0.000426607	23.33009709	1.435501654	6	7	positive regulation of cholesterol esterification
GO:0019226	0.000426607	23.33009709	1.435501654	6	7	transmission of nerve impulse
GO:0019433	0.000426607	23.33009709	1.435501654	6	7	triglyceride catabolic process
GO:0034368	0.000426607	23.33009709	1.435501654	6	7	protein-lipid complex remodeling
GO:0034369	0.000426607	23.33009709	1.435501654	6	7	plasma lipoprotein particle remodeling
GO:0034370	0.000426607	23.33009709	1.435501654	6	7	triglyceride-rich lipoprotein particle remodeling
GO:0034372	0.000426607	23.33009709	1.435501654	6	7	very-low-density lipoprotein particle remodeling
GO:0034377	0.000426607	23.33009709	1.435501654	6	7	plasma lipoprotein particle assembly
GO:0034380	0.000426607	23.33009709	1.435501654	6	7	high-density lipoprotein particle assembly
GO:0034433	0.000426607	23.33009709	1.435501654	6	7	steroid esterification
GO:0034434	0.000426607	23.33009709	1.435501654	6	7	sterol esterification
GO:0034435	0.000426607	23.33009709	1.435501654	6	7	cholesterol esterification
GO:0045940	0.000426607	23.33009709	1.435501654	6	7	positive regulation of steroid metabolic process
GO:0065005	0.000426607	23.33009709	1.435501654	6	7	protein-lipid complex assembly
GO:0071825	0.000426607	23.33009709	1.435501654	6	7	protein-lipid complex subunit organization
GO:0071827	0.000426607	23.33009709	1.435501654	6	7	plasma lipoprotein particle organization
GO:0015850	0.000435126	3.31606077	7.587651599	17	37	organic hydroxy compound transport
GO:0016051	0.000435126	3.31606077	7.587651599	17	37	carbohydrate biosynthetic process
GO:0009135	0.000458103	3.466859484	6.972436604	16	34	purine nucleoside diphosphate metabolic process
GO:0009179	0.000458103	3.466859484	6.972436604	16	34	purine ribonucleoside diphosphate metabolic process
GO:0009185	0.000458103	3.466859484	6.972436604	16	34	ribonucleoside diphosphate metabolic process
GO:1903034	0.000458103	3.466859484	6.972436604	16	34	regulation of response to wounding
GO:1903046	0.000458103	3.466859484	6.972436604	16	34	meiotic cell cycle process
GO:0072329	0.000468067	4.221891355	5.126791621	13	25	monocarboxylic acid catabolic process
GO:0009132	0.000488386	2.891706924	9.638368247	20	47	nucleoside diphosphate metabolic process
GO:0044255	0.0005206	1.583879221	58.65049614	82	286	cellular lipid metabolic process
GO:1901698	0.00063706	2.158605599	18.45644983	32	90	response to nitrogen compound
GO:0006066	0.000643795	2.345335516	14.76515987	27	72	alcohol metabolic process
GO:0007126	0.000724157	3.43998087	6.562293275	15	32	meiotic nuclear division
GO:0006575	0.000728004	2.603191875	11.27894157	22	55	cellular modified amino acid metabolic process
GO:0008202	0.000728004	2.603191875	11.27894157	22	55	steroid metabolic process

GO:0071310	0.000729627	1.47236097	80.79823594	107	394	cellular response to organic substance
GO:1901136	0.000754523	2.85162746	9.228224917	19	45	carbohydrate derivative catabolic process
GO:0055082	0.000792708	2.12172236	18.6615215	32	91	cellular chemical homeostasis
GO:0007017	0.000844675	1.708361382	38.55347299	57	188	microtubule-based process
GO:0042060	0.000883513	1.957738749	23.37816979	38	114	wound healing
GO:0022402	0.000892664	1.614879955	48.60198456	69	237	cell cycle process
GO:0050900	0.000918057	3.013762147	7.997794928	17	39	leukocyte migration
GO:0006941	0.000941481	5.837925446	3.076074972	9	15	striated muscle contraction
GO:0005996	0.000974472	2.403552874	12.91951488	24	63	monosaccharide metabolic process
