# Supplementary material for: Short bowel syndrome results in increased gene expression associated with proliferation, inflammation, bile acid synthesis and immune system activation: RNA sequencing a zebrafish SBS model
Source: BMC Genomics. 2017 Jan 25;18:23. doi: 10.1186/s12864-016-3433-4 (PMC5264326; doi:10.1186/s12864-016-3433-4)
Supplement: Additional file 4: Table S3. — GO enrichment analysis for biological processes under-represented by genes of SBS zebrafish intestine as compared to sham. (PDF 135 kb) [file 12864_2016_3433_MOESM4_ESM.pdf]

GOBPID	Pvalue	OddsRatio	ExpCount	Count	Size	Term
GO:0044260	1.24E-36	0.478735086	636.952591	413	3106	cellular macromolecule metabolic process
GO:0006396	1.43E-27	0.064201134	72.18522602	6	352	RNA processing
GO:0010467	1.45E-25	0.460526499	352.3131202	203	1718	gene expression
GO:0043170	1.07E-23	0.572155481	693.7574421	511	3383	macromolecule metabolic process
GO:0016070	8.47E-21	0.476308467	298.9944873	174	1458	RNA metabolic process
GO:0090304	3.12E-19	0.515961705	340.0088203	213	1658	nucleic acid metabolic process
GO:0044267	2.74E-18	0.526930861	340.8291069	217	1662	cellular protein metabolic process
GO:0034641	4.66E-17	0.58732995	467.3583241	332	2279	cellular nitrogen compound metabolic process
GO:0034660	7.19E-17	0.039987242	39.16868798	2	191	ncRNA metabolic process
GO:0034470	1.99E-16	0	31.99117971	0	156	ncRNA processing
GO:0016071	7.69E-16	0.075152256	42.03969129	4	205	mRNA metabolic process
GO:0006139	9.97E-16	0.579664318	394.3528115	273	1923	nucleobase-containing compound metabolic process
GO:0022613	6.77E-15	0.093990962	42.24476295	5	206	ribonucleoprotein complex biogenesis
GO:0034645	9.20E-14	0.5786084	327.4994487	223	1597	cellular macromolecule biosynthetic process
GO:0046483	2.94E-13	0.616163221	406.862183	295	1984	heterocycle metabolic process
GO:0009059	2.99E-13	0.587571671	330.7805954	228	1613	macromolecule biosynthetic process
GO:0044237	5.75E-13	0.684700281	822.5424476	687	4011	cellular metabolic process
GO:0016192	8.66E-13	0.290030898	74.23594267	26	362	vesicle-mediated transport
GO:0043412	8.92E-13	0.547551538	245.2657111	157	1196	macromolecule modification
GO:0006807	2.48E-12	0.652112453	494.4277839	379	2411	nitrogen compound metabolic process
GO:0006397	2.52E-12	0.093174847	34.24696803	4	167	mRNA processing
GO:0006725	6.29E-12	0.635582406	407.477398	302	1987	cellular aromatic compound metabolic process
GO:0046907	1.06E-11	0.359835485	88.79603087	38	433	intracellular transport
GO:0042254	2.07E-11	0.079837771	29.94046307	3	146	ribosome biogenesis
GO:0043043	1.38E-10	0.304224088	63.16207277	23	308	peptide biosynthetic process
GO:0006412	1.49E-10	0.298463297	61.52149945	22	300	translation
GO:0006464	1.88E-10	0.580663189	231.9360529	155	1131	cellular protein modification process
GO:0036211	1.88E-10	0.580663189	231.9360529	155	1131	protein modification process
GO:0051641	2.31E-10	0.472609476	127.3495039	70	621	cellular localization
GO:0019538	2.37E-10	0.657286953	396.1984564	300	1932	protein metabolic process
GO:0016072	2.99E-10	0	19.48180816	0	95	rRNA metabolic process
GO:0045184	3.24E-10	0.388172471	85.30981257	39	416	establishment of protein localization
GO:0015031	3.89E-10	0.381724967	82.2337376	37	401	protein transport

GO:0044271	4.12E-10	0.636006142	323.3980154	236	1577	cellular nitrogen compound biosynthetic process
GO:0006364	4.76E-10	0	19.07166483	0	93	rRNA processing
GO:1901360	4.92E-10	0.669234525	419.5766262	323	2046	organic cyclic compound metabolic process
GO:0008104	1.29E-09	0.442528158	100.8952591	52	492	protein localization
GO:0008380	1.56E-09	0.115562957	27.88974642	4	136	RNA splicing
GO:0051649	1.61E-09	0.468093728	112.3792723	61	548	establishment of localization in cell
GO:0006518	1.99E-09	0.358569625	68.49393605	29	334	peptide metabolic process
GO:0043604	2.30E-09	0.35980123	68.28886439	29	333	amide biosynthetic process
GO:0044238	3.04E-09	0.73480353	837.3076075	726	4083	primary metabolic process
GO:1902582	4.27E-09	0.330719825	58.65049614	23	286	single-organism intracellular transport
GO:0070727	2.24E-08	0.408914712	73.41565601	35	358	cellular macromolecule localization
GO:0006399	2.92E-08	0.043535136	18.25137817	1	89	tRNA metabolic process
GO:0034613	3.35E-08	0.412928082	72.80044101	35	355	cellular protein localization
GO:0006886	8.52E-08	0.365435599	55.9845645	24	273	intracellular protein transport
GO:0044249	8.91E-08	0.708010763	405.8368247	324	1979	cellular biosynthetic process
GO:0071840	9.52E-08	0.693959289	353.3384785	276	1723	cellular component organization or biogenesis
GO:0043632	1.53E-07	0.302017465	41.83461963	15	204	modification-dependent macromolecule catabolic process
GO:0000375	2.03E-07	0.116039229	20.91730981	3	102	RNA splicing, via transesterification reactions
GO:0000377	2.48E-07	0.117239788	20.71223815	3	101	RNA splicing, via transesterification reactions with bulged adenosir nucleophile
GO:0000398	2.48E-07	0.117239788	20.71223815	3	101	mRNA splicing, via spliceosome
GO:0019941	3.37E-07	0.310445387	40.8092613	15	199	modification-dependent protein catabolic process
GO:0033036	4.22E-07	0.553152499	119.7618523	75	584	macromolecule localization
GO:0044265	4.24E-07	0.406924995	59.26571114	28	289	cellular macromolecule catabolic process
GO:0006511	5.39E-07	0.315725643	40.19404631	15	196	ubiquitin-dependent protein catabolic process
GO:0016482	8.78E-07	0.24917201	30.14553473	9	147	cytoplasmic transport
GO:0016568	1.03E-06	0.302395447	36.29768467	13	177	chromatin modification
GO:0009451	1.25E-06	0	12.09922822	0	59	RNA modification
GO:0071704	1.37E-06	0.781049486	867.2480706	777	4229	organic substance metabolic process
GO:0009058	1.57E-06	0.740634468	428.8048512	354	2091	biosynthetic process
GO:1901576	1.76E-06	0.739443962	417.7309813	344	2037	organic substance biosynthetic process
GO:0070647	1.99E-06	0.415292835	54.13891951	26	264	protein modification by small protein conjugation or removal
GO:0071826	2.01E-06	0.159707256	20.50716648	4	100	ribonucleoprotein complex subunit organization
GO:0008033	2.51E-06	0	11.48401323	0	56	tRNA processing
GO:0061024	3.57E-06	0.366998206	42.03969129	18	205	membrane organization

GO:0022618	4.35E-06	0.166744753	19.68687982	4	96	ribonucleoprotein complex assembly
GO:0006325	4.76E-06	0.371071397	41.62954796	18	203	chromatin organization
GO:0006401	5.02E-06	0	10.86879824	0	53	RNA catabolic process
GO:0007005	5.07E-06	0.193607231	21.32745314	5	104	mitochondrion organization
GO:0016569	8.81E-06	0.199741689	20.71223815	5	101	covalent chromatin modification
GO:0032774	1.17E-05	0.692307895	217.3759647	166	1060	RNA biosynthetic process
GO:0016570	1.27E-05	0.204048862	20.30209482	5	99	histone modification
GO:0097659	1.35E-05	0.69407987	216.9658214	166	1058	nucleic acid-templated transcription
GO:0048193	1.47E-05	0.111402318	14.5600882	2	71	Golgi vesicle transport
GO:0006351	1.55E-05	0.695859809	216.5556781	166	1056	transcription, DNA-templated
GO:0043603	1.60E-05	0.541689674	80.38809261	49	392	cellular amide metabolic process
GO:0034654	2.31E-05	0.71886942	250.8026461	198	1223	nucleobase-containing compound biosynthetic process
GO:0044802	2.62E-05	0.389013617	37.73318633	17	184	single-organism membrane organization
GO:0006402	3.18E-05	0	9.228224917	0	45	mRNA catabolic process
GO:0044085	3.29E-05	0.656882771	147.6515987	107	720	cellular component biogenesis
GO:0006996	3.29E-05	0.700052561	204.2513782	157	996	organelle organization
GO:0010498	3.32E-05	0.326558249	28.71003308	11	140	proteasomal protein catabolic process
GO:0006457	3.76E-05	0.218145062	19.07166483	5	93	protein folding
GO:0043161	4.51E-05	0.331794586	28.29988975	11	138	proteasome-mediated ubiquitin-dependent protein catabolic proces
GO:0051171	5.18E-05	0.725017114	238.2932745	189	1162	regulation of nitrogen compound metabolic process
GO:0016043	5.24E-05	0.75818871	330.5755237	274	1612	cellular component organization
GO:2000112	5.47E-05	0.719940187	225.9889746	178	1102	regulation of cellular macromolecule biosynthetic process
GO:0060255	5.94E-05	0.748701682	293.4575524	240	1431	regulation of macromolecule metabolic process
GO:1901566	6.09E-05	0.633880121	117.0959206	82	571	organonitrogen compound biosynthetic process
GO:0032446	6.20E-05	0.458169935	45.93605292	24	224	protein modification by small protein conjugation
GO:0010468	6.91E-05	0.72852846	236.2425579	188	1152	regulation of gene expression
GO:0044257	7.93E-05	0.462929953	45.52590959	24	222	cellular protein catabolic process
GO:0009057	8.75E-05	0.541942044	65.82800441	40	321	macromolecule catabolic process
GO:0010556	9.12E-05	0.728854699	227.629548	181	1110	regulation of macromolecule biosynthetic process
GO:0000956	0.000100798	0	8.202866593	0	40	nuclear-transcribed mRNA catabolic process
GO:0051603	0.000101183	0.467787115	45.11576626	24	220	proteolysis involved in cellular protein catabolic process
GO:0019438	0.000120428	0.746666667	258.3902977	210	1260	aromatic compound biosynthetic process
GO:0051252	0.000131892	0.728284024	214.2998897	170	1045	regulation of RNA metabolic process
GO:0043933	0.000133233	0.664101777	128.9900772	94	629	macromolecular complex subunit organization

GO:0018130	0.000150655	0.751165739	260.8511577	213	1272	heterocycle biosynthetic process
GO:1902580	0.000215688	0.490113882	45.11576626	25	220	single-organism cellular localization
GO:0019219	0.000221279	0.741758242	223.5281147	180	1090	regulation of nucleobase-containing compound metabolic process
GO:0030163	0.000236669	0.509461426	48.80705623	28	238	protein catabolic process
GO:0031326	0.000238217	0.74788658	234.1918412	190	1142	regulation of cellular biosynthetic process
GO:0019222	0.000262779	0.778987294	321.1422271	271	1566	regulation of metabolic process
GO:0018193	0.00028066	0.502054349	45.93605292	26	224	peptidyl-amino acid modification
GO:0007034	0.000319233	0	7.177508269	0	35	vacuolar transport
GO:0031123	0.000319233	0	7.177508269	0	35	RNA 3'-end processing
GO:2001141	0.000333027	0.741938979	208.9680265	168	1019	regulation of RNA biosynthetic process
GO:1903506	0.000353282	0.742928661	208.7629548	168	1018	regulation of nucleic acid-templated transcription
GO:0080090	0.00037277	0.77700664	294.0727674	247	1434	regulation of primary metabolic process
GO:0006355	0.000397312	0.744915027	208.3528115	168	1016	regulation of transcription, DNA-templated
GO:0090501	0.000401978	0	6.972436604	0	34	RNA phosphodiester bond hydrolysis
GO:0016567	0.000416671	0.499147485	42.65490628	24	208	protein ubiquitination
GO:0009889	0.000417697	0.758308024	235.4222712	193	1148	regulation of biosynthetic process
GO:0042274	0.000497496	0.089658106	9.023153252	1	44	ribosomal small subunit biogenesis
GO:0009987	0.000606516	0.832131792	1314.509372	1257	6410	cellular process
GO:0006497	0.000656847	0.154144241	10.66372657	2	52	protein lipidation
GO:0031323	0.000676044	0.789057858	301.2502756	256	1469	regulation of cellular metabolic process
GO:0043543	0.000797845	0.15731201	10.45865491	2	51	protein acylation
GO:0006473	0.000802433	0	6.35722161	0	31	protein acetylation
GO:0006888	0.000802433	0	6.35722161	0	31	ER to Golgi vesicle-mediated transport
GO:0009247	0.000802433	0	6.35722161	0	31	glycolipid biosynthetic process
GO:0016197	0.000880745	0.20638126	12.09922822	3	59	endosomal transport
