# Supplementary material for: Short bowel syndrome results in increased gene expression associated with proliferation, inflammation, bile acid synthesis and immune system activation: RNA sequencing a zebrafish SBS model
Source: BMC Genomics. 2017 Jan 25;18:23. doi: 10.1186/s12864-016-3433-4 (PMC5264326; doi:10.1186/s12864-016-3433-4)
Supplement: Additional file 5: Table S4. — IPA analysis of percentage of up- and downregulated genes within each significant pathway. (PDF 295 kb) [file 12864_2016_3433_MOESM5_ESM.pdf]

Ingenuity Canonical Pathways	-log(p-value)	Ratio	Downregulated	Upregulated
FXR/RXR Activation	1.62E01	2.99E-01	13/127 (10%)	25/127 (20%)
LXR/RXR Activation	1.45E01	2.89E-01	8/121 (7%)	27/121 (22%)
Coagulation System	1.4E01	5.43E-01	0/35 (0%)	19/35 (54%)
Acute Phase Response Signaling	1.12E01	2.19E-01	3/169 (2%)	34/169 (20%)
Extrinsic Prothrombin Activation Pathway	9.88E00	6.88E-01	0/16 (0%)	11/16 (69%)
Superpathway of Cholesterol Biosynthesis	9.8E00	5E-01	0/28 (0%)	14/28 (50%)
LPS/IL-1 Mediated Inhibition of RXR Function	7.33E00	1.64E-01	22/219 (10%)	14/219 (6%)
Intrinsic Prothrombin Activation Pathway	7.32E00	4.14E-01	0/29 (0%)	12/29 (41%)
Folate Polyglutamylation	6.08E00	1E00	4/5 (80%)	1/5 (20%)
Glycolysis I	6.03E00	4E-01	5/25 (20%)	5/25 (20%)
Complement System	5.96E00	3.24E-01	0/37 (0%)	12/37 (32%)
Folate Transformations I	5.45E00	6.67E-01	4/9 (44%)	2/9 (22%)
Cholesterol Biosynthesis I	5.43E00	5.38E-01	0/13 (0%)	7/13 (54%)
Cholesterol Biosynthesis II (via 24,25-dihydrolanosterol)	5.43E00	5.38E-01	0/13 (0%)	7/13 (54%)
Cholesterol Biosynthesis III (via Desmosterol)	5.43E00	5.38E-01	0/13 (0%)	7/13 (54%)
Mitotic Roles of Polo-Like Kinase	5.12E00	2.27E-01	1/66 (2%)	14/66 (21%)
Estrogen Biosynthesis	5.1E00	2.97E-01	8/37 (22%)	3/37 (8%)
Gluconeogenesis I	5.03E00	3.6E-01	6/25 (24%)	3/25 (12%)
TR/RXR Activation	4.93E00	2E-01	10/85 (12%)	7/85 (8%)
Cell Cycle: G2/M DNA Damage Checkpoint Regulation	4.56E00	2.45E-01	1/49 (2%)	11/49 (22%)
Superpathway of Geranylgeranyldiphosphate Biosynthesis I (via Mevalonate)	4.46E00	4.12E-01	0/17 (0%)	7/17 (41%)
Histidine Degradation III	4.4E00	6.25E-01	1/8 (13%)	4/8 (50%)
PXR/RXR Activation	4.38E00	2.09E-01	11/67 (16%)	3/67 (4%)
Fatty Acid β-oxidation I	4.31E00	3E-01	8/30 (27%)	1/30 (3%)
Mevalonate Pathway I	4.23E00	4.62E-01	0/13 (0%)	6/13 (46%)
Tyrosine Degradation I	4.19E00	8E-01	1/5 (20%)	3/5 (60%)
Leukocyte Extravasation Signaling	4.15E00	1.36E-01	5/198 (3%)	22/198 (11%)
Retinol Biosynthesis	3.95E00	2.73E-01	4/33 (12%)	5/33 (15%)
Glioma Invasiveness Signaling	3.87E00	2.11E-01	1/57 (2%)	11/57 (19%)
The Visual Cycle	3.81E00	4E-01	3/15 (20%)	3/15 (20%)
Role of Tissue Factor in Cancer	3.47E00	1.55E-01	2/110 (2%)	15/110 (14%)
γ-linolenate Biosynthesis II (Animals)	3.46E00	3.53E-01	5/17 (29%)	1/17 (6%)

EIF2 Signaling	3.43E00	1.3E-01	22/185 (12%)	2/185 (1%)
Superpathway of Serine and Glycine Biosynthesis I	3.39E00	5.71E-01	2/7 (29%)	2/7 (29%)
Regulation of eIF4 and p70S6K Signaling	3.26E00	1.37E-01	13/146 (9%)	7/146 (5%)
Production of Nitric Oxide and Reactive Oxygen Species in Macrophages	3.22E00	1.28E-01	6/180 (3%)	17/180 (9%)
Glutathione Redox Reactions I	3.17E00	3.16E-01	4/19 (21%)	2/19 (11%)
Fatty Acid Activation	3.15E00	3.85E-01	4/13 (31%)	1/13 (8%)
Airway Pathology in Chronic Obstructive Pulmonary Disease	3.11E00	5E-01	1/8 (13%)	3/8 (38%)
Hepatic Cholestasis	3.07E00	1.3E-01	10/162 (6%)	11/162 (7%)
Tryptophan Degradation III (Eukaryotic)	3.03E00	3E-01	4/20 (20%)	2/20 (10%)
Stearate Biosynthesis I (Animals)	3.01E00	2.29E-01	5/35 (14%)	3/35 (9%)
Isoleucine Degradation I	2.98E00	3.57E-01	3/14 (21%)	2/14 (14%)
IL-12 Signaling and Production in Macrophages	2.86E00	1.33E-01	5/135 (4%)	13/135 (10%)
Maturity Onset Diabetes of Young (MODY) Signaling	2.79E00	2.73E-01	5/22 (23%)	1/22 (5%)
Tetrahydrofolate Salvage from 5,10-methenyltetrahydrofolate	2.69E00	6E-01	1/5 (20%)	2/5 (40%)
Citrulline-Nitric Oxide Cycle	2.69E00	6E-01	0/5 (0%)	3/5 (60%)
VDR/RXR Activation	2.6E00	1.54E-01	8/78 (10%)	4/78 (5%)
Cyclins and Cell Cycle Regulation	2.6E00	1.54E-01	1/78 (1%)	11/78 (14%)
Estrogen-mediated S-phase Entry	2.58E00	2.5E-01	1/24 (4%)	5/24 (21%)
ATM Signaling	2.56E00	1.69E-01	1/59 (2%)	9/59 (15%)
Mitochondrial L-carnitine Shuttle Pathway	2.56E00	2.94E-01	4/17 (24%)	1/17 (6%)
Putrescine Degradation III	2.56E00	2.94E-01	4/17 (24%)	1/17 (6%)
Glutaryl-CoA Degradation	2.5E00	3.64E-01	3/11 (27%)	1/11 (9%)
Circadian Rhythm Signaling	2.49E00	2.12E-01	6/33 (18%)	1/33 (3%)
Role of JAK family kinases in IL-6-type Cytokine Signaling	2.49E00	2.4E-01	0/25 (0%)	6/25 (24%)
Bupropion Degradation	2.49E00	2.4E-01	5/25 (20%)	1/25 (4%)
Atherosclerosis Signaling	2.46E00	1.29E-01	3/124 (2%)	13/124 (10%)
Epoxysqualene Biosynthesis	2.43E00	1E00	0/2 (0%)	2/2 (100%)
Glycine Biosynthesis III	2.43E00	1E00	0/2 (0%)	2/2 (100%)
Glycine Biosynthesis I	2.43E00	1E00	2/2 (100%)	0/2 (0%)
Arginine Biosynthesis IV	2.41E00	5E-01	1/6 (17%)	2/6 (33%)
Zymosterol Biosynthesis	2.41E00	5E-01	0/6 (0%)	3/6 (50%)
Superpathway of Melatonin Degradation	2.4E00	1.61E-01	8/62 (13%)	2/62 (3%)
Acetone Degradation I (to Methylglyoxal)	2.4E00	2.31E-01	5/26 (19%)	1/26 (4%)
Endothelin-1 Signaling	2.39E00	1.16E-01	6/172 (3%)	14/172 (8%)

IL-8 Signaling	2.39E00	1.14E-01	3/184 (2%)	18/184 (10%)
Xenobiotic Metabolism Signaling	2.36E00	1.03E-01	17/271 (6%)	11/271 (4%)
Role of Pattern Recognition Receptors in Recognition of Bacteria and Viruses	2.35E00	1.26E-01	2/127 (2%)	14/127 (11%)
ILK Signaling	2.33E00	1.13E-01	5/186 (3%)	16/186 (9%)
GADD45 Signaling	2.33E00	2.63E-01	1/19 (5%)	4/19 (21%)
Colorectal Cancer Metastasis Signaling	2.3E00	1.06E-01	3/236 (1%)	22/236 (9%)
Eicosanoid Signaling	2.3E00	1.56E-01	1/64 (2%)	9/64 (14%)
RAR Activation	2.23E00	1.11E-01	13/190 (7%)	8/190 (4%)
Sertoli Cell-Sertoli Cell Junction Signaling	2.23E00	1.12E-01	1/178 (1%)	19/178 (11%)
Bile Acid Biosynthesis, Neutral Pathway	2.2E00	3.08E-01	2/13 (15%)	2/13 (15%)
Melatonin Degradation I	2.15E00	1.58E-01	7/57 (12%)	2/57 (4%)
Tec Kinase Signaling	2.12E00	1.14E-01	7/158 (4%)	11/158 (7%)
Colanic Acid Building Blocks Biosynthesis	2.08E00	2.86E-01	2/14 (14%)	2/14 (14%)
Signaling by Rho Family GTPases	2.06E00	1.03E-01	6/234 (3%)	18/234 (8%)
Germ Cell-Sertoli Cell Junction Signaling	2.06E00	1.12E-01	3/160 (2%)	15/160 (9%)
Clathrin-mediated Endocytosis Signaling	2.04E00	1.08E-01	7/185 (4%)	13/185 (7%)
Polyamine Regulation in Colon Cancer	2.04E00	2.27E-01	2/22 (9%)	3/22 (14%)
Pyrimidine Deoxyribonucleotides De Novo Biosynthesis I	2.04E00	2.27E-01	2/22 (9%)	3/22 (14%)
Hepatic Fibrosis / Hepatic Stellate Cell Activation	2.03E00	1.06E-01	2/198 (1%)	19/198 (10%)
Thyronamine and Iodothyronamine Metabolism	1.97E00	6.67E-01	1/3 (33%)	1/3 (33%)
Thyroid Hormone Metabolism I (via Deiodination)	1.97E00	6.67E-01	1/3 (33%)	1/3 (33%)
mTOR Signaling	1.97E00	1.06E-01	14/188 (7%)	6/188 (3%)
MIF Regulation of Innate Immunity	1.96E00	1.71E-01	1/41 (2%)	6/41 (15%)
Type II Diabetes Mellitus Signaling	1.94E00	1.2E-01	10/117 (9%)	4/117 (3%)
Retinoate Biosynthesis I	1.87E00	1.82E-01	4/33 (12%)	2/33 (6%)
Nicotine Degradation II	1.87E00	1.43E-01	8/63 (13%)	1/63 (2%)
Fatty Acid α-oxidation	1.86E00	2.5E-01	3/16 (19%)	1/16 (6%)
Prostanoid Biosynthesis	1.84E00	3.33E-01	0/9 (0%)	3/9 (33%)
Sucrose Degradation V (Mammalian)	1.84E00	3.33E-01	1/9 (11%)	2/9 (22%)
Ketolysis	1.84E00	3.33E-01	1/9 (11%)	2/9 (22%)
Axonal Guidance Signaling	1.82E00	8.78E-02	10/433 (2%)	28/433 (6%)
Nicotine Degradation III	1.8E00	1.48E-01	7/54 (13%)	1/54 (2%)
Bladder Cancer Signaling	1.79E00	1.26E-01	4/87 (5%)	7/87 (8%)
TGF-β Signaling	1.79E00	1.26E-01	3/87 (3%)	8/87 (9%)

Triacylglycerol Biosynthesis	1.75E00	1.71E-01	4/35 (11%)	2/35 (6%)
Acute Myeloid Leukemia Signaling	1.75E00	1.3E-01	2/77 (3%)	8/77 (10%)
RhoGDI Signaling	1.74E00	1.04E-01	6/173 (3%)	12/173 (7%)
Actin Nucleation by ARP-WASP Complex	1.71E00	1.43E-01	0/56 (0%)	8/56 (14%)
Ketogenesis	1.71E00	3E-01	1/10 (10%)	2/10 (20%)
Histidine Degradation VI	1.71E00	3E-01	1/10 (10%)	2/10 (20%)
Uracil Degradation II (Reductive)	1.69E00	5E-01	0/4 (0%)	2/4 (50%)
Spermine and Spermidine Degradation I	1.69E00	5E-01	1/4 (25%)	1/4 (25%)
Thymine Degradation	1.69E00	5E-01	0/4 (0%)	2/4 (50%)
Rapoport-Luebering Glycolytic Shunt	1.69E00	5E-01	1/4 (25%)	1/4 (25%)
HIF1α Signaling	1.68E00	1.18E-01	3/102 (3%)	9/102 (9%)
Tryptophan Degradation X (Mammalian, via Tryptamine)	1.68E00	2.22E-01	4/18 (22%)	0/18 (0%)
Valine Degradation I	1.68E00	2.22E-01	3/18 (17%)	1/18 (6%)
Regulation of Cellular Mechanics by Calpain Protease	1.67E00	1.4E-01	1/57 (2%)	7/57 (12%)
Agranulocyte Adhesion and Diapedesis	1.66E00	1.01E-01	2/189 (1%)	17/189 (9%)
Regulation of Actin-based Motility by Rho	1.66E00	1.21E-01	2/91 (2%)	9/91 (10%)
Aryl Hydrocarbon Receptor Signaling	1.63E00	1.07E-01	9/140 (6%)	6/140 (4%)
Agrin Interactions at Neuromuscular Junction	1.63E00	1.3E-01	2/69 (3%)	7/69 (10%)
Role of MAPK Signaling in the Pathogenesis of Influenza	1.63E00	1.3E-01	2/69 (3%)	7/69 (10%)
Phospholipases	1.63E00	1.38E-01	1/58 (2%)	7/58 (12%)
Actin Cytoskeleton Signaling	1.62E00	9.68E-02	7/217 (3%)	14/217 (6%)
Salvage Pathways of Pyrimidine Ribonucleotides	1.59E00	1.18E-01	3/93 (3%)	8/93 (9%)
Synaptic Long Term Depression	1.58E00	1.06E-01	6/142 (4%)	9/142 (6%)
NRF2-mediated Oxidative Stress Response	1.58E00	1E-01	8/180 (4%)	10/180 (6%)
Role of Osteoblasts, Osteoclasts and Chondrocytes in Rheumatoid Arthritis	1.58E00	9.59E-02	5/219 (2%)	16/219 (7%)
Inhibition of Matrix Metalloproteases	1.54E00	1.54E-01	0/39 (0%)	6/39 (15%)
phagosome maturation	1.51E00	1.08E-01	1/120 (1%)	12/120 (10%)
Regulation of the Epithelial-Mesenchymal Transition Pathway	1.5E00	9.78E-02	4/184 (2%)	14/184 (8%)
Serine Biosynthesis	1.49E00	4E-01	0/5 (0%)	2/5 (40%)
Trans, trans-farnesyl Diphosphate Biosynthesis	1.49E00	4E-01	0/5 (0%)	2/5 (40%)
dTMP De Novo Biosynthesis	1.49E00	4E-01	2/5 (40%)	0/5 (0%)
Glutamate Degradation III (via 4-aminobutyrate)	1.49E00	4E-01	1/5 (20%)	1/5 (20%)
Glutathione-mediated Detoxification	1.48E00	1.67E-01	4/30 (13%)	1/30 (3%)
FGF Signaling	1.48E00	1.18E-01	5/85 (6%)	5/85 (6%)

Estrogen-Dependent Breast Cancer Signaling	1.47E00	1.29E-01	4/62 (6%)	4/62 (6%)
RhoA Signaling	1.46E00	1.07E-01	3/122 (2%)	10/122 (8%)
p53 Signaling	1.45E00	1.12E-01	4/98 (4%)	7/98 (7%)
phagosome formation	1.43E00	1.08E-01	2/111 (2%)	10/111 (9%)
Pyridoxal 5'-phosphate Salvage Pathway	1.4E00	1.25E-01	3/64 (5%)	5/64 (8%)
Serotonin Degradation	1.4E00	1.25E-01	7/64 (11%)	1/64 (2%)
Semaphorin Signaling in Neurons	1.39E00	1.32E-01	2/53 (4%)	5/53 (9%)
Histamine Degradation	1.39E00	2.31E-01	3/13 (23%)	0/13 (0%)
BMP signaling pathway	1.39E00	1.18E-01	3/76 (4%)	6/76 (8%)
Granulocyte Adhesion and Diapedesis	1.38E00	9.6E-02	1/177 (1%)	16/177 (9%)
Superpathway of Methionine Degradation	1.37E00	1.56E-01	5/32 (16%)	0/32 (0%)
Virus Entry via Endocytic Pathways	1.36E00	1.12E-01	6/89 (7%)	4/89 (4%)
PAK Signaling	1.36E00	1.12E-01	2/89 (2%)	8/89 (9%)
Urea Cycle	1.33E00	3.33E-01	0/6 (0%)	2/6 (33%)
Glycine Cleavage Complex	1.33E00	3.33E-01	2/6 (33%)	0/6 (0%)
Glycogen Biosynthesis II (from UDP-D-Glucose)	1.33E00	3.33E-01	1/6 (17%)	1/6 (17%)
GDP-mannose Biosynthesis	1.33E00	3.33E-01	1/6 (17%)	1/6 (17%)
MIF-mediated Glucocorticoid Regulation	1.32E00	1.52E-01	1/33 (3%)	4/33 (12%)
Superpathway of Citrulline Metabolism	1.31E00	2.14E-01	0/14 (0%)	3/14 (21%)
Hereditary Breast Cancer Signaling	1.3E00	1.01E-01	4/129 (3%)	9/129 (7%)
Rac Signaling	1.29E00	1.06E-01	2/104 (2%)	9/104 (9%)
CCR3 Signaling in Eosinophils	1.28E00	1.03E-01	5/117 (4%)	7/117 (6%)
Inhibition of Angiogenesis by TSP1	1.28E00	1.47E-01	1/34 (3%)	4/34 (12%)
Remodeling of Epithelial Adherens Junctions	1.27E00	1.18E-01	2/68 (3%)	6/68 (9%)
Macropinocytosis Signaling	1.27E00	1.18E-01	2/68 (3%)	6/68 (9%)
IL-22 Signaling	1.26E00	1.67E-01	0/24 (0%)	4/24 (17%)
Dopamine Degradation	1.26E00	1.67E-01	3/24 (13%)	1/24 (4%)
Pancreatic Adenocarcinoma Signaling	1.24E00	1.04E-01	1/106 (1%)	10/106 (9%)
Molecular Mechanisms of Cancer	1.24E00	8.22E-02	10/365 (3%)	20/365 (5%)
3-phosphoinositide Degradation	1.23E00	9.66E-02	5/145 (3%)	9/145 (6%)
MSP-RON Signaling Pathway	1.23E00	1.3E-01	2/46 (4%)	4/46 (9%)
Cell Cycle Regulation by BTG Family Proteins	1.23E00	1.43E-01	2/35 (6%)	3/35 (9%)
Thyroid Hormone Metabolism II (via Conjugation and/or Degradation)	1.23E00	1.43E-01	3/35 (9%)	2/35 (6%)
Noradrenaline and Adrenaline Degradation	1.23E00	1.43E-01	5/35 (14%)	0/35 (0%)

Androgen Biosynthesis	1.23E00	2E-01	2/15 (13%)	1/15 (7%)
L-cysteine Degradation II	1.22E00	1E00	1/1 (100%)	0/1 (0%)
Lanosterol Biosynthesis	1.22E00	1E00	0/1 (0%)	1/1 (100%)
4-hydroxybenzoate Biosynthesis	1.22E00	1E00	0/1 (0%)	1/1 (100%)
4-hydroxyphenylpyruvate Biosynthesis	1.22E00	1E00	0/1 (0%)	1/1 (100%)
Mouse Embryonic Stem Cell Pluripotency	1.2E00	1.05E-01	2/95 (2%)	8/95 (8%)
Phosphatidylcholine Biosynthesis I	1.2E00	2.86E-01	2/7 (29%)	0/7 (0%)
Adenine and Adenosine Salvage III	1.2E00	2.86E-01	1/7 (14%)	1/7 (14%)
Aspartate Degradation II	1.2E00	2.86E-01	2/7 (29%)	0/7 (0%)
ERK/MAPK Signaling	1.2E00	9.09E-02	9/187 (5%)	8/187 (4%)
AMPK Signaling	1.17E00	9.46E-02	7/148 (5%)	7/148 (5%)
Breast Cancer Regulation by Stathmin1	1.13E00	8.9E-02	5/191 (3%)	12/191 (6%)
Prolactin Signaling	1.13E00	1.1E-01	4/73 (5%)	4/73 (5%)
STAT3 Pathway	1.13E00	1.1E-01	2/73 (3%)	6/73 (8%)
CDK5 Signaling	1.11E00	1.01E-01	3/99 (3%)	7/99 (7%)
CXCR4 Signaling	1.1E00	9.21E-02	5/152 (3%)	9/152 (6%)
Tryptophan Degradation to 2-amino-3-carboxymuconate Semialdehyde	1.09E00	2.5E-01	1/8 (13%)	1/8 (13%)
Purine Ribonucleosides Degradation to Ribose-1-phosphate	1.09E00	2.5E-01	1/8 (13%)	1/8 (13%)
D-myo-inositol (1,4,5,6)-Tetrakisphosphate Biosynthesis	1.05E00	9.38E-02	4/128 (3%)	8/128 (6%)
D-myo-inositol (3,4,5,6)-tetrakisphosphate Biosynthesis	1.05E00	9.38E-02	4/128 (3%)	8/128 (6%)
HER-2 Signaling in Breast Cancer	1.05E00	1.05E-01	2/76 (3%)	6/76 (8%)
Phosphatidylglycerol Biosynthesis II (Non-plastidic)	1.03E00	1.67E-01	3/18 (17%)	0/18 (0%)
Cell Cycle: G1/S Checkpoint Regulation	1.03E00	1.09E-01	2/64 (3%)	5/64 (8%)
Thyroid Cancer Signaling	1.03E00	1.25E-01	3/40 (8%)	2/40 (5%)
IL-6 Signaling	1.02E00	9.48E-02	1/116 (1%)	10/116 (9%)
D-myo-inositol-5-phosphate Metabolism	1E00	9.03E-02	4/144 (3%)	9/144 (6%)
Phosphatidylethanolamine Biosynthesis II	9.99E-01	2.22E-01	1/9 (11%)	1/9 (11%)
Calcium Transport I	9.99E-01	2.22E-01	1/9 (11%)	1/9 (11%)
Leucine Degradation I	9.99E-01	2.22E-01	1/9 (11%)	1/9 (11%)
Heme Biosynthesis II	9.99E-01	2.22E-01	1/9 (11%)	1/9 (11%)
UDP-N-acetyl-D-galactosamine Biosynthesis II	9.99E-01	2.22E-01	1/9 (11%)	1/9 (11%)
Dopamine Receptor Signaling	9.96E-01	1.03E-01	5/78 (6%)	3/78 (4%)
FcγRIIB Signaling in B Lymphocytes	9.94E-01	1.22E-01	1/41 (2%)	4/41 (10%)
DNA damage-induced 14-3-3σ Signaling	9.78E-01	1.58E-01	0/19 (0%)	3/19 (16%)

Cysteine Biosynthesis III (mammalia)	9.78E-01	1.58E-01	3/19 (16%)	0/19 (0%)
Reelin Signaling in Neurons	9.72E-01	1.01E-01	1/79 (1%)	7/79 (9%)
Epithelial Adherens Junction Signaling	9.66E-01	8.9E-02	1/146 (1%)	12/146 (8%)
Ephrin Receptor Signaling	9.6E-01	8.62E-02	4/174 (2%)	11/174 (6%)
Ceramide Signaling	9.48E-01	1E-01	4/80 (5%)	4/80 (5%)
HMGB1 Signaling	9.44E-01	9.17E-02	1/120 (1%)	10/120 (8%)
Glucocorticoid Receptor Signaling	9.39E-01	8E-02	7/275 (3%)	15/275 (5%)
Role of CHK Proteins in Cell Cycle Checkpoint Control	9.36E-01	1.09E-01	1/55 (2%)	5/55 (9%)
Asparagine Degradation I	9.28E-01	5E-01	1/2 (50%)	0/2 (0%)
UDP-D-xylose and UDP-D-glucuronate Biosynthesis	9.28E-01	5E-01	1/2 (50%)	0/2 (0%)
β-alanine Degradation I	9.28E-01	5E-01	1/2 (50%)	0/2 (0%)
Proline Degradation	9.28E-01	5E-01	1/2 (50%)	0/2 (0%)
Alanine Degradation III	9.28E-01	5E-01	0/2 (0%)	1/2 (50%)
Alanine Biosynthesis II	9.28E-01	5E-01	0/2 (0%)	1/2 (50%)
L-cysteine Degradation III	9.28E-01	5E-01	1/2 (50%)	0/2 (0%)
4-hydroxyproline Degradation I	9.28E-01	5E-01	1/2 (50%)	0/2 (0%)
Sulfate Activation for Sulfonation	9.28E-01	5E-01	1/2 (50%)	0/2 (0%)
Anandamide Degradation	9.28E-01	5E-01	1/2 (50%)	0/2 (0%)
Glycine Degradation (Creatine Biosynthesis)	9.28E-01	5E-01	1/2 (50%)	0/2 (0%)
Cysteine Biosynthesis/Homocysteine Degradation	9.28E-01	5E-01	1/2 (50%)	0/2 (0%)
Cardiolipin Biosynthesis II	9.28E-01	5E-01	1/2 (50%)	0/2 (0%)
Putrescine Biosynthesis III	9.28E-01	5E-01	0/2 (0%)	1/2 (50%)
Formaldehyde Oxidation II (Glutathione-dependent)	9.28E-01	5E-01	1/2 (50%)	0/2 (0%)
Glutamate Biosynthesis II	9.28E-01	5E-01	1/2 (50%)	0/2 (0%)
Glutamate Degradation X	9.28E-01	5E-01	1/2 (50%)	0/2 (0%)
Glutamate Dependent Acid Resistance	9.28E-01	5E-01	0/2 (0%)	1/2 (50%)
Purine Nucleotides Degradation II (Aerobic)	9.26E-01	1.5E-01	1/20 (5%)	2/20 (10%)
Glycine Betaine Degradation	9.2E-01	2E-01	2/10 (20%)	0/10 (0%)
Thrombin Signaling	9.16E-01	8.38E-02	5/191 (3%)	11/191 (6%)
EGF Signaling	9.08E-01	1.07E-01	2/56 (4%)	4/56 (7%)
Growth Hormone Signaling	9.02E-01	1.01E-01	3/69 (4%)	4/69 (6%)
Renin-Angiotensin Signaling	8.97E-01	9.17E-02	3/109 (3%)	7/109 (6%)
Role of NANOG in Mammalian Embryonic Stem Cell Pluripotency	8.6E-01	9.01E-02	4/111 (4%)	6/111 (5%)
Ethanol Degradation II	8.59E-01	1.21E-01	4/33 (12%)	0/33 (0%)

Small Cell Lung Cancer Signaling	8.55E-01	9.86E-02	4/71 (6%)	3/71 (4%)
Phospholipase C Signaling	8.5E-01	7.95E-02	5/239 (2%)	14/239 (6%)
Antioxidant Action of Vitamin C	8.49E-01	9.18E-02	2/98 (2%)	7/98 (7%)
Basal Cell Carcinoma Signaling	8.32E-01	9.72E-02	3/72 (4%)	4/72 (6%)
IL-9 Signaling	8.25E-01	1.18E-01	2/34 (6%)	2/34 (6%)
Oncostatin M Signaling	8.25E-01	1.18E-01	0/34 (0%)	4/34 (12%)
3-phosphoinositide Biosynthesis	8.09E-01	8.33E-02	5/156 (3%)	8/156 (5%)
α-Adrenergic Signaling	7.99E-01	9.2E-02	4/87 (5%)	4/87 (5%)
FAK Signaling	7.99E-01	9.2E-02	3/87 (3%)	5/87 (6%)
Mitochondrial Dysfunction	7.99E-01	8.19E-02	9/171 (5%)	5/171 (3%)
Paxillin Signaling	7.93E-01	8.91E-02	3/101 (3%)	6/101 (6%)
Role of JAK2 in Hormone-like Cytokine Signaling	7.92E-01	1.14E-01	1/35 (3%)	3/35 (9%)
Toll-like Receptor Signaling	7.89E-01	9.46E-02	1/74 (1%)	6/74 (8%)
TREM1 Signaling	7.68E-01	9.33E-02	0/75 (0%)	7/75 (9%)
L-carnitine Biosynthesis	7.65E-01	3.33E-01	1/3 (33%)	0/3 (0%)
Diphthamide Biosynthesis	7.65E-01	3.33E-01	1/3 (33%)	0/3 (0%)
Trehalose Degradation II (Trehalase)	7.65E-01	3.33E-01	0/3 (0%)	1/3 (33%)
NADH Repair	7.65E-01	3.33E-01	1/3 (33%)	0/3 (0%)
Tetrahydrobiopterin Biosynthesis I	7.65E-01	3.33E-01	0/3 (0%)	1/3 (33%)
Glutathione Redox Reactions II	7.65E-01	3.33E-01	0/3 (0%)	1/3 (33%)
Methionine Salvage II (Mammalian)	7.65E-01	3.33E-01	1/3 (33%)	0/3 (0%)
Thyroid Hormone Biosynthesis	7.65E-01	3.33E-01	0/3 (0%)	1/3 (33%)
Tetrahydrobiopterin Biosynthesis II	7.65E-01	3.33E-01	0/3 (0%)	1/3 (33%)
Glutamate Degradation II	7.65E-01	3.33E-01	1/3 (33%)	0/3 (0%)
S-adenosyl-L-methionine Biosynthesis	7.65E-01	3.33E-01	1/3 (33%)	0/3 (0%)
Tyrosine Biosynthesis IV	7.65E-01	3.33E-01	0/3 (0%)	1/3 (33%)
4-aminobutyrate Degradation I	7.65E-01	3.33E-01	1/3 (33%)	0/3 (0%)
Fatty Acid β-oxidation III (Unsaturated, Odd Number)	7.65E-01	3.33E-01	1/3 (33%)	0/3 (0%)
Aspartate Biosynthesis	7.65E-01	3.33E-01	1/3 (33%)	0/3 (0%)
GM-CSF Signaling	7.59E-01	9.68E-02	1/62 (2%)	5/62 (8%)
Triacylglycerol Degradation	7.53E-01	1.25E-01	0/24 (0%)	3/24 (13%)
Antiproliferative Role of Somatostatin Receptor 2	7.37E-01	9.52E-02	4/63 (6%)	2/63 (3%)
Ubiquinol-10 Biosynthesis (Eukaryotic)	7.32E-01	1.54E-01	1/13 (8%)	1/13 (8%)
Choline Biosynthesis III	7.32E-01	1.54E-01	1/13 (8%)	1/13 (8%)

Oleate Biosynthesis II (Animals)	7.32E-01	1.54E-01	2/13 (15%)	0/13 (0%)
p70S6K Signaling	7.26E-01	8.4E-02	4/119 (3%)	6/119 (5%)
HGF Signaling	7.24E-01	8.57E-02	2/105 (2%)	7/105 (7%)
Role of BRCA1 in DNA Damage Response	7.09E-01	8.97E-02	0/78 (0%)	7/78 (9%)
Calcium Signaling	7.08E-01	7.87E-02	5/178 (3%)	9/178 (5%)
Non-Small Cell Lung Cancer Signaling	6.94E-01	9.23E-02	4/65 (6%)	2/65 (3%)
CNTF Signaling	6.82E-01	9.62E-02	1/52 (2%)	4/52 (8%)
DNA Double-Strand Break Repair by Homologous Recombination	6.82E-01	1.43E-01	0/14 (0%)	2/14 (14%)
Phenylalanine Degradation IV (Mammalian, via Side Chain)	6.82E-01	1.43E-01	2/14 (14%)	0/14 (0%)
Antiproliferative Role of TOB in T Cell Signaling	6.81E-01	1.15E-01	1/26 (4%)	2/26 (8%)
IL-15 Signaling	6.74E-01	9.09E-02	1/66 (2%)	5/66 (8%)
Angiopoietin Signaling	6.74E-01	9.09E-02	2/66 (3%)	4/66 (6%)
PI3K/AKT Signaling	6.67E-01	8.13E-02	3/123 (2%)	7/123 (6%)
Tight Junction Signaling	6.63E-01	7.78E-02	2/167 (1%)	11/167 (7%)
Cdc42 Signaling	6.63E-01	7.78E-02	3/167 (2%)	10/167 (6%)
Fc Epsilon RI Signaling	6.61E-01	8.26E-02	3/109 (3%)	6/109 (6%)
Retinoate Biosynthesis II	6.54E-01	2.5E-01	1/4 (25%)	0/4 (0%)
Creatine-phosphate Biosynthesis	6.54E-01	2.5E-01	0/4 (0%)	1/4 (25%)
2-ketoglutarate Dehydrogenase Complex	6.54E-01	2.5E-01	1/4 (25%)	0/4 (0%)
Heme Degradation	6.54E-01	2.5E-01	0/4 (0%)	1/4 (25%)
Heme Biosynthesis from Uroporphyrinogen-III I	6.54E-01	2.5E-01	1/4 (25%)	0/4 (0%)
Catecholamine Biosynthesis	6.54E-01	2.5E-01	1/4 (25%)	0/4 (0%)
Eumelanin Biosynthesis	6.54E-01	2.5E-01	1/4 (25%)	0/4 (0%)
α-tocopherol Degradation	6.54E-01	2.5E-01	0/4 (0%)	1/4 (25%)
Myo-inositol Biosynthesis	6.54E-01	2.5E-01	0/4 (0%)	1/4 (25%)
Geranylgeranyldiphosphate Biosynthesis	6.54E-01	2.5E-01	0/4 (0%)	1/4 (25%)
Proline Biosynthesis I	6.54E-01	2.5E-01	0/4 (0%)	1/4 (25%)
Melatonin Degradation II	6.54E-01	2.5E-01	1/4 (25%)	0/4 (0%)
Arginine Degradation I (Arginase Pathway)	6.54E-01	2.5E-01	1/4 (25%)	0/4 (0%)
L-cysteine Degradation I	6.54E-01	2.5E-01	1/4 (25%)	0/4 (0%)
Phenylalanine Degradation I (Aerobic)	6.54E-01	2.5E-01	0/4 (0%)	1/4 (25%)
Neuroprotective Role of THOP1 in Alzheimer's Disease	6.49E-01	1E-01	1/40 (3%)	3/40 (8%)
Type I Diabetes Mellitus Signaling	6.46E-01	8.18E-02	3/110 (3%)	6/110 (5%)
Role of IL-17A in Arthritis	6.38E-01	9.26E-02	1/54 (2%)	4/54 (7%)

Chondroitin Sulfate Biosynthesis	6.38E-01	9.26E-02	2/54 (4%)	3/54 (6%)
NAD biosynthesis II (from tryptophan)	6.37E-01	1.33E-01	1/15 (7%)	1/15 (7%)
γ-glutamyl Cycle	6.37E-01	1.33E-01	1/15 (7%)	1/15 (7%)
Corticotropin Releasing Hormone Signaling	6.31E-01	8.11E-02	4/111 (4%)	5/111 (5%)
Gap Junction Signaling	6.3E-01	7.74E-02	6/155 (4%)	6/155 (4%)
Huntington's Disease Signaling	6.26E-01	7.39E-02	7/230 (3%)	10/230 (4%)
IGF-1 Signaling	6.25E-01	8.25E-02	3/97 (3%)	5/97 (5%)
Thrombopoietin Signaling	6.17E-01	9.09E-02	3/55 (5%)	2/55 (4%)
Integrin Signaling	6.15E-01	7.46E-02	3/201 (1%)	12/201 (6%)
Melanoma Signaling	6E-01	9.52E-02	1/42 (2%)	3/42 (7%)
UVC-Induced MAPK Signaling	6E-01	9.52E-02	1/42 (2%)	3/42 (7%)
CDP-diacylglycerol Biosynthesis I	5.95E-01	1.25E-01	2/16 (13%)	0/16 (0%)
Oxidative Ethanol Degradation III	5.95E-01	1.25E-01	2/16 (13%)	0/16 (0%)
Parkinson's Signaling	5.95E-01	1.25E-01	0/16 (0%)	2/16 (13%)
Pyrimidine Ribonucleotides Interconversion	5.89E-01	1.03E-01	1/29 (3%)	2/29 (7%)
Caveolar-mediated Endocytosis Signaling	5.81E-01	8.45E-02	3/71 (4%)	3/71 (4%)
T Helper Cell Differentiation	5.81E-01	8.45E-02	0/71 (0%)	6/71 (8%)
ErbB2-ErbB3 Signaling	5.77E-01	8.77E-02	2/57 (4%)	3/57 (5%)
Heparan Sulfate Biosynthesis	5.77E-01	8.77E-02	2/57 (4%)	3/57 (5%)
Dermatan Sulfate Biosynthesis	5.77E-01	8.77E-02	2/57 (4%)	3/57 (5%)
Ceramide Biosynthesis	5.7E-01	2E-01	0/5 (0%)	1/5 (20%)
Tetrapyrrole Biosynthesis II	5.7E-01	2E-01	0/5 (0%)	1/5 (20%)
Galactose Degradation I (Leloir Pathway)	5.7E-01	2E-01	0/5 (0%)	1/5 (20%)
Lactose Degradation III	5.7E-01	2E-01	0/5 (0%)	1/5 (20%)
IL-17 Signaling	5.64E-01	8.33E-02	1/72 (1%)	5/72 (7%)
JAK/Stat Signaling	5.64E-01	8.33E-02	2/72 (3%)	4/72 (6%)
Sonic Hedgehog Signaling	5.62E-01	1E-01	0/30 (0%)	3/30 (10%)
Myc Mediated Apoptosis Signaling	5.58E-01	8.62E-02	3/58 (5%)	2/58 (3%)
RAN Signaling	5.57E-01	1.18E-01	0/17 (0%)	2/17 (12%)
Adenosine Nucleotides Degradation II	5.57E-01	1.18E-01	1/17 (6%)	1/17 (6%)
Methionine Degradation I (to Homocysteine)	5.57E-01	1.18E-01	2/17 (12%)	0/17 (0%)
iNOS Signaling	5.56E-01	9.09E-02	0/44 (0%)	4/44 (9%)
Dermatan Sulfate Biosynthesis (Late Stages)	5.56E-01	9.09E-02	2/44 (5%)	2/44 (5%)
14-3-3-mediated Signaling	5.5E-01	7.69E-02	2/117 (2%)	7/117 (6%)

Gα12/13 Signaling	5.5E-01	7.69E-02	1/117 (1%)	8/117 (7%)
p38 MAPK Signaling	5.5E-01	7.69E-02	4/117 (3%)	5/117 (4%)
Cardiac Hypertrophy Signaling	5.48E-01	7.17E-02	5/223 (2%)	11/223 (5%)
LPS-stimulated MAPK Signaling	5.47E-01	8.22E-02	2/73 (3%)	4/73 (5%)
NF-κB Activation by Viruses	5.47E-01	8.22E-02	2/73 (3%)	4/73 (5%)
Ephrin B Signaling	5.47E-01	8.22E-02	2/73 (3%)	4/73 (5%)
Superpathway of Inositol Phosphate Compounds	5.43E-01	7.25E-02	5/193 (3%)	9/193 (5%)
Protein Ubiquitination Pathway	5.37E-01	7.06E-02	3/255 (1%)	15/255 (6%)
Pyrimidine Ribonucleotides De Novo Biosynthesis	5.36E-01	9.68E-02	1/31 (3%)	2/31 (6%)
FLT3 Signaling in Hematopoietic Progenitor Cells	5.31E-01	8.11E-02	1/74 (1%)	5/74 (7%)
Human Embryonic Stem Cell Pluripotency	5.25E-01	7.46E-02	5/134 (4%)	5/134 (4%)
Chondroitin Sulfate Biosynthesis (Late Stages)	5.14E-01	8.7E-02	2/46 (4%)	2/46 (4%)
Relaxin Signaling	5.14E-01	7.41E-02	5/135 (4%)	5/135 (4%)
Proline Biosynthesis II (from Arginine)	5.03E-01	1.67E-01	0/6 (0%)	1/6 (17%)
Pyruvate Fermentation to Lactate	5.03E-01	1.67E-01	0/6 (0%)	1/6 (17%)
Serotonin and Melatonin Biosynthesis	5.03E-01	1.67E-01	1/6 (17%)	0/6 (0%)
Arginine Degradation VI (Arginase 2 Pathway)	5.03E-01	1.67E-01	0/6 (0%)	1/6 (17%)
Thioredoxin Pathway	5.03E-01	1.67E-01	0/6 (0%)	1/6 (17%)
Selenocysteine Biosynthesis II (Archaea and Eukaryotes)	5.03E-01	1.67E-01	1/6 (17%)	0/6 (0%)
UDP-N-acetyl-D-glucosamine Biosynthesis II	5.03E-01	1.67E-01	0/6 (0%)	1/6 (17%)
Ceramide Degradation	5.03E-01	1.67E-01	1/6 (17%)	0/6 (0%)
VEGF Family Ligand-Receptor Interactions	5E-01	7.89E-02	3/76 (4%)	3/76 (4%)
Communication between Innate and Adaptive Immune Cells	4.99E-01	7.69E-02	2/91 (2%)	5/91 (5%)
Ethanol Degradation IV	4.91E-01	1.05E-01	2/19 (11%)	0/19 (0%)
Factors Promoting Cardiogenesis in Vertebrates	4.86E-01	7.61E-02	4/92 (4%)	3/92 (3%)
Ephrin A Signaling	4.76E-01	8.33E-02	2/48 (4%)	2/48 (4%)
fMLP Signaling in Neutrophils	4.75E-01	7.41E-02	3/108 (3%)	5/108 (5%)
Fcγ Receptor-mediated Phagocytosis in Macrophages and Monocytes	4.73E-01	7.53E-02	4/93 (4%)	3/93 (3%)
Chronic Myeloid Leukemia Signaling	4.73E-01	7.53E-02	2/93 (2%)	5/93 (5%)
ERK5 Signaling	4.72E-01	7.94E-02	1/63 (2%)	4/63 (6%)
Role of JAK1 and JAK3 in γc Cytokine Signaling	4.72E-01	7.94E-02	1/63 (2%)	4/63 (6%)
Sphingosine-1-phosphate Signaling	4.63E-01	7.34E-02	3/109 (3%)	5/109 (5%)
Gαs Signaling	4.63E-01	7.34E-02	4/109 (4%)	4/109 (4%)
Cardiomyocyte Differentiation via BMP Receptors	4.61E-01	1E-01	1/20 (5%)	1/20 (5%)

Superoxide Radicals Degradation	4.49E-01	1.43E-01	0/7 (0%)	1/7 (14%)
Glycoaminoglycan-protein Linkage Region Biosynthesis	4.49E-01	1.43E-01	0/7 (0%)	1/7 (14%)
Pregnenolone Biosynthesis	4.49E-01	1.43E-01	1/7 (14%)	0/7 (0%)
GDP-glucose Biosynthesis	4.49E-01	1.43E-01	0/7 (0%)	1/7 (14%)
Glioma Signaling	4.47E-01	7.37E-02	2/95 (2%)	5/95 (5%)
Role of Macrophages, Fibroblasts and Endothelial Cells in Rheumatoid Arthritis	4.47E-01	6.71E-02	3/298 (1%)	17/298 (6%)
CD40 Signaling	4.42E-01	7.69E-02	1/65 (2%)	4/65 (6%)
Heparan Sulfate Biosynthesis (Late Stages)	4.41E-01	8E-02	2/50 (4%)	2/50 (4%)
Adipogenesis pathway	4.34E-01	7.09E-02	4/127 (3%)	5/127 (4%)
Estrogen Receptor Signaling	4.34E-01	7.09E-02	5/127 (4%)	4/127 (3%)
GABA Receptor Signaling	4.13E-01	7.46E-02	3/67 (4%)	2/67 (3%)
Endometrial Cancer Signaling	4.09E-01	7.69E-02	2/52 (4%)	2/52 (4%)
Sphingosine and Sphingosine-1-phosphate Metabolism	4.04E-01	1.25E-01	1/8 (13%)	0/8 (0%)
Glucose and Glucose-1-phosphate Degradation	4.04E-01	1.25E-01	0/8 (0%)	1/8 (13%)
Salvage Pathways of Pyrimidine Deoxyribonucleotides	4.04E-01	1.25E-01	0/8 (0%)	1/8 (13%)
Sphingomyelin Metabolism	4.04E-01	1.25E-01	1/8 (13%)	0/8 (0%)
IL-10 Signaling	4E-01	7.35E-02	0/68 (0%)	5/68 (7%)
Ovarian Cancer Signaling	3.95E-01	6.87E-02	1/131 (1%)	8/131 (6%)
UVB-Induced MAPK Signaling	3.93E-01	7.55E-02	2/53 (4%)	2/53 (4%)
Gαq Signaling	3.93E-01	6.8E-02	5/147 (3%)	5/147 (3%)
CCR5 Signaling in Macrophages	3.86E-01	7.25E-02	2/69 (3%)	3/69 (4%)
TCA Cycle II (Eukaryotic)	3.85E-01	8.7E-02	2/23 (9%)	0/23 (0%)
Cholecystokinin/Gastrin-mediated Signaling	3.79E-01	6.93E-02	1/101 (1%)	6/101 (6%)
Unfolded protein response	3.79E-01	7.41E-02	1/54 (2%)	3/54 (6%)
Cardiac β-adrenergic Signaling	3.76E-01	6.77E-02	5/133 (4%)	4/133 (3%)
ErbB Signaling	3.71E-01	6.98E-02	3/86 (3%)	3/86 (3%)
PEDF Signaling	3.61E-01	7.04E-02	2/71 (3%)	3/71 (4%)
Chemokine Signaling	3.61E-01	7.04E-02	1/71 (1%)	4/71 (6%)
B Cell Activating Factor Signaling	3.54E-01	7.5E-02	0/40 (0%)	3/40 (8%)
Transcriptional Regulatory Network in Embryonic Stem Cells	3.54E-01	7.5E-02	1/40 (3%)	2/40 (5%)
Wnt/Ca+ pathway	3.51E-01	7.14E-02	1/56 (2%)	3/56 (5%)
Neuregulin Signaling	3.49E-01	6.82E-02	3/88 (3%)	3/88 (3%)
G Beta Gamma Signaling	3.49E-01	6.82E-02	3/88 (3%)	3/88 (3%)

Altered T Cell and B Cell Signaling in Rheumatoid Arthritis	3.49E-01	6.82E-02	0/88 (0%)	6/88 (7%)
Wnt/β-catenin Signaling	3.44E-01	6.51E-02	2/169 (1%)	9/169 (5%)
IL-17A Signaling in Gastric Cells	3.42E-01	8E-02	0/25 (0%)	2/25 (8%)
Sperm Motility	3.41E-01	6.61E-02	4/121 (3%)	4/121 (3%)
Glutamate Receptor Signaling	3.38E-01	7.02E-02	2/57 (4%)	2/57 (4%)
IL-1 Signaling	3.19E-01	6.59E-02	2/91 (2%)	4/91 (4%)
G-Protein Coupled Receptor Signaling	3.08E-01	6.25E-02	7/256 (3%)	9/256 (4%)
Serotonin Receptor Signaling	2.96E-01	6.82E-02	2/44 (5%)	1/44 (2%)
PDGF Signaling	2.95E-01	6.49E-02	3/77 (4%)	2/77 (3%)
SAPK/JNK Signaling	2.91E-01	6.38E-02	2/94 (2%)	4/94 (4%)
PPAR Signaling	2.91E-01	6.38E-02	4/94 (4%)	2/94 (2%)
Androgen Signaling	2.87E-01	6.31E-02	3/111 (3%)	4/111 (4%)
Role of RIG1-like Receptors in Antiviral Innate Immunity	2.83E-01	6.67E-02	1/45 (2%)	2/45 (4%)
Acyl-CoA Hydrolysis	2.76E-01	8.33E-02	1/12 (8%)	0/12 (0%)
Purine Nucleotides De Novo Biosynthesis II	2.76E-01	8.33E-02	0/12 (0%)	1/12 (8%)
Mineralocorticoid Biosynthesis	2.76E-01	8.33E-02	1/12 (8%)	0/12 (0%)
Dendritic Cell Maturation	2.75E-01	6.15E-02	3/179 (2%)	8/179 (4%)
Glioblastoma Multiforme Signaling	2.74E-01	6.16E-02	2/146 (1%)	7/146 (5%)
Role of Oct4 in Mammalian Embryonic Stem Cell Pluripotency	2.71E-01	6.52E-02	1/46 (2%)	2/46 (4%)
PCP pathway	2.69E-01	6.35E-02	0/63 (0%)	4/63 (6%)
nNOS Signaling in Neurons	2.59E-01	6.38E-02	1/47 (2%)	2/47 (4%)
Retinoic acid Mediated Apoptosis Signaling	2.59E-01	6.25E-02	3/64 (5%)	1/64 (2%)
IL-17A Signaling in Airway Cells	2.59E-01	6.25E-02	1/64 (2%)	3/64 (5%)
Amyotrophic Lateral Sclerosis Signaling	2.57E-01	6.12E-02	2/98 (2%)	4/98 (4%)
NAD Phosphorylation and Dephosphorylation	2.53E-01	7.69E-02	0/13 (0%)	1/13 (8%)
Glucocorticoid Biosynthesis	2.53E-01	7.69E-02	1/13 (8%)	0/13 (0%)
4-1BB Signaling in T Lymphocytes	2.44E-01	6.45E-02	0/31 (0%)	2/31 (6%)
Urate Biosynthesis/Inosine 5'-phosphate Degradation	2.33E-01	7.14E-02	0/14 (0%)	1/14 (7%)
Vitamin-C Transport	2.33E-01	7.14E-02	0/14 (0%)	1/14 (7%)
Cytotoxic T Lymphocyte-mediated Apoptosis of Target Cells	2.31E-01	6.25E-02	2/32 (6%)	0/32 (0%)
Telomere Extension by Telomerase	2.15E-01	6.67E-02	0/15 (0%)	1/15 (7%)
Chondroitin Sulfate Degradation (Metazoa)	2.15E-01	6.67E-02	0/15 (0%)	1/15 (7%)
Leukotriene Biosynthesis	2.15E-01	6.67E-02	0/15 (0%)	1/15 (7%)
Granzyme B Signaling	1.98E-01	6.25E-02	1/16 (6%)	0/16 (0%)

Mismatch Repair in Eukaryotes	1.98E-01	6.25E-02	0/16 (0%)	1/16 (6%)
Dermatan Sulfate Degradation (Metazoa)	1.98E-01	6.25E-02	0/16 (0%)	1/16 (6%)
